# Genetic analysis of children with congenital ocular anomalies in three ecological regions of Nepal: a phase II of Nepal pediatric ocular diseases study

**DOI:** 10.1186/s12881-020-01116-9

**Published:** 2020-09-22

**Authors:** Srijana Adhikari, Neelam Thakur, Ujjowala Shrestha, Mohan K Shrestha, Murarai Manshrestha, Bijay Thapa, Manish Poudel, Ajaya Kunwar

**Affiliations:** 1 Tilganga Institute of Ophthalmology, PO Box 561, Kathmandu, Nepal; 2 National Academy of Medical Sciences NAMS, Bir Hospital, New Delhi, India; 3 Sudrishti Eye Clinic, Kathmandu, Nepal; 4 Patan Academy of Health Sciences, Patan, Nepal; 5 The Kathmandu Centre for Genomics and Research Laboratory, Kathmandu, Nepal

**Keywords:** Nepal, Pediatric, Genetics, Congenital anomalies, Ocular

## Abstract

**Background:**

Genetic eye diseases constitute a large and heterogeneous group of childhood ocular morbidity. Individual diseases may cause multiple structural anomalies and developmental features. Nepal Pediatric Ocular Disease Study (NPODS) was a population-based epidemiological study conducted across three ecological regions of Nepal to determine the prevalence and etiology of childhood ocular morbidity and blindness. In Phase II of this study, genetic analysis was performed for children who were found to have congenital ocular anomalies.

**Method:**

It was a cross sectional descriptive study. A total of 10,270 children across three different ecological regions in Nepal (Low lands, hills, and mountains) underwent ocular examinations in NPODS. Out of 374 (3.6%) of children with ocular abnormalities, 30 were thought to be congenital in nature. Targeted genetic analysis, including genotyping for genes specific to presenting phenotype, was performed for 25 children using serum samples.

**Results:**

Out of 25 children, 18 had meaningful genetic results. Analysis revealed one missense alteration G12411T of Zinc Finger Homeobox 4 (*ZFHX4)* gene in one participant among 10 with congenital ptosis and another missense variation T > C P. Y374 C of Signaling Receptor and Transporter Retinol 6 (*STRA6)* gene in one participant among 3 with microphthalmos.

**Conclusion:**

The study is first of its kind from Nepal and mutant genes were unique to Nepalese Population. Further analysis of genetic factors is crucial to better understand genetic association with ocular diseases and conditions. This helps further in genetic counseling and probably gene therapy to prevent blindness from these conditions.

## Background

Visual impairment is one of the most common disabilities affecting children. There is an estimated 1.4 million children worldwide who are blind, two-thirds of whom live in developing countries such as Nepal [1]. Controlling childhood blindness has been a top priority of the World Health Organization since its launch of the VISION 2020: The Right to Sight global initiative in 1999 [2].

Congenital ocular anomalies are one of the important causes of childhood ocular morbidity and blindness. Out of approximately 4000 genetic diseases and syndromes which affect humans, at least one third involves the eye [3, 4]. The genetic and hereditary eye diseases account for 11–39% of childhood blindness with more common in developed than in the developing world [5] Treatment is unfortunately often difficult or nonexistent for many of these congenital anomalies. There is a limited understanding of these diseases and there is lack of clinical trial and testing of different therapies [6, 7].. Genetic testing in these situations can help to confirm diagnoses and thus provide prognostic information, guide interventions for the child, and assist in counseling regarding risk of disease occurrence in subsequent children. Pediatric Ophthalmologists are the ones who are on front line in assessing the patients and their families in these situation. There should be a good understanding and knowledge of various genetic tests indicated in different disease. The Genetic Eye Disease Task Force of the American Academy of Ophthalmology has recommended various genetic testing of different congenital ocular anomalies [8]. .

Phase I of Nepal Pediatric Ocular Disease Study (NPODS) determined the prevalence of childhood ocular morbidity and blindness across three ecological regions (lowlands, hills, mountains) of Nepal [9]. The three ecological regions were selected to represent Nepalese population with different socioeconomic and geographic background. The prevalence of childhood blindness was found to be 0.067% with congenital and hereditary ocular diseases accounting for 50% of blindness in children. Another study, a nationwide blind school survey done in Nepal found 30% of children suffering from hereditary diseases [10]. This burden of childhood blindness due to these congenital diseases is high. Managing these diseases becomes even more challenging in developing countries like Nepal due to lack of good infrastructure for clinical trial and tests. There is a handful of studies carried out in Nepal on genetic analysis of ocular diseases in adult populations [11]. There is no published study of such analysis in children. The Phase II of NPODS was carried out to find out risk factors of ocular morbidities identified in the first study [12]. In this Phase II study, we also conducted genetic analysis in children with congenital anomalies to understand the genetic implications of these diseases in Nepalese children. Looking at the frequency of mutation or allele in this type of congenital anomalies would help in future direction on diagnosis and management of these diseases in Nepalese population.

## Method

**Fig. 1:**
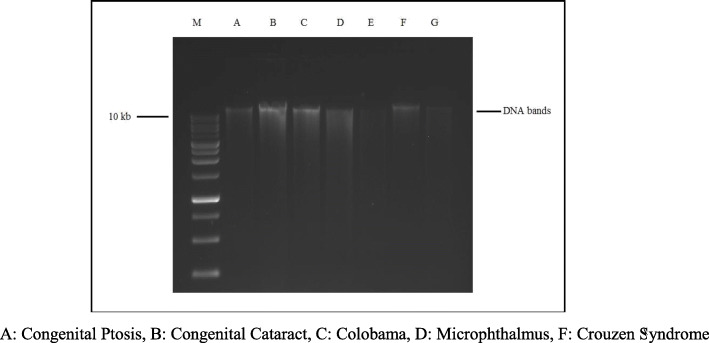
Agarose Gel electrophoresis

**Fig. 2:**
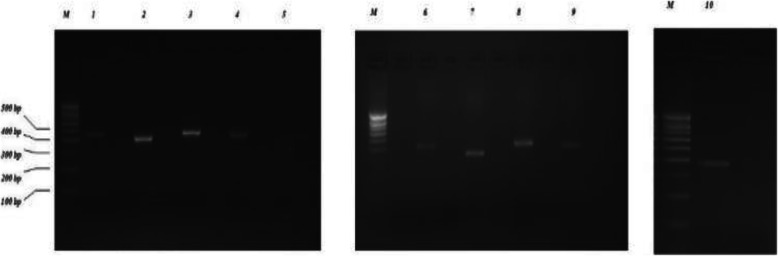
Agarose Gel electrophoresis of PCR products on 2% (w/V) agarose gel

This study was an expansion investigation from the phase I of NPODS. Children aged 0 to 16 years were included in the study. Congenital and hereditary ocular diseases were diagnosed by detailed history and clinical examination by Pediatric Ophthalmologists. After getting pre informed written consent at the time of examination, the children’s parents were instructed to report to primary health center for blood sample collection. Children who did not show up for the sample collection and those who did not give consent were excluded from the study The genetic test was carried out in Kathmandu Centre for Genomics and Research Laboratory (KCGRL) in Kathmandu Nepal. A single 5 ml venous blood sample was collected from each child while awake. DNA was extracted from the blood samples using a commercially-available DNA Extraction Kit (Qiagen; city, state) as per manufacturer’s protocol and after evaluation for quality. The extracted DNA was checked for the quality control through 1% agarose gel electrophoresis using ethidium bromide as a staining dye. Genotyping was done for a selected number of specific genes which corresponded to the presenting phenotype of each child. (Fig. 1) Locations of mutations were identified by conventional methods as well as by available reference Single Nucleotide Polymorphism (SNP) ID. Genomic reference sequences, spanning the mutation locus were downloaded from db SNP as well as from Genbank database. Polymerase chain reaction primers to amplify these loci were designed using Primer design version 3 online tool. Primers were chosen such that they amplify 300–500 bp size amplicons and location of mutation is central to the amplicon with Sanger sequencing reads having high QV bars. Additional file 1 shows the primers designed to amplify each loci. In silico validation of these primer pairs was performed using NCBI Primer BLAST tool. Furthermore, Primers were designed using Chromosomal DNA reference sequence, db SNP reference sequences and primer 3 design software available at bioinfo.ut.ee/primer3–0.4.0. (Additional file 1) The Polymerase Chain Reaction (PCR) was carried out in a 25 μl volume with the final mix containing 10× PCR buffer, 1.25 mM dNTPs, 25 mM MgCl_2_, 10 pmole of each primer, 2.5 U of *Taq* polymerase and 2.5 μl of DNA template. The sample was heated to 95 °C for 5 min, followed by 35 cycles of 95 °C for 30 s, 55 °C for 30s, 72 °C for 30 s and a final extension at 72 °C for 10mins**.** (Fig. 2).

Through the confirmation from correct size of amplification, samples were further subjected to DNA sequencing**.** This DNA sequencing analysis was carried out to determine the precise order of nucleotides of given DNA molecule. It was used to determine the sequence of individual genes. (Additional file 2). Cycle sequencing reaction for each sample was performed in 0.2 mL PCR tube. The reaction included Terminator Ready Reaction Mix (BigDye®) Terminator v3.1 Cycle Sequencing Kit, Applied Biosystems) (0.5 μL), BigDye® Sequencing Buffer (1.8 μL), One sequencing primer (3.2 pmol), and template DNA (2 μL) with Standard Milli Q (SMQ) water (4.7 μL) to make up the volume of 10.0 μL. For each sample two sequencing reactions were performed; one using forward primer and other with reverse primer. The cycle sequencing protocol was as follows: Initial denaturation at 96 °C for 1 min, followed by 25 cycles of 96 °C for 10 s, annealing at 50 °C for 5 s, and elongation at 60 °C for 4 min**.** (Additional file 3).

### Data management and analysis

Data were entered into the electronic database The codes, recodes, consistency, outlines etc. were assessed through the use of Microsoft Excel. Data analysis was done in Statistical Package for Social Science (SPSS) version16. For the association of the categorical data, Chi Square test was used. *P* value < 0.005 was considered as statistically significant.

## Results

A total of 10,270 children, aged 0 to 16 years across three different ecological regions of Nepal underwent vision screening Within this cohort, 5208 children (50.7%) were from Lowland, 3136 (30.5%) were from the hill, and 1926 (18.8%) were from the mountain region. Ocular abnormalities were present in 374 (3.6%) of all the children examined. The ocular abnormalities in 30 of these children were thought to be congenital in nature.

**Fig. 3:**
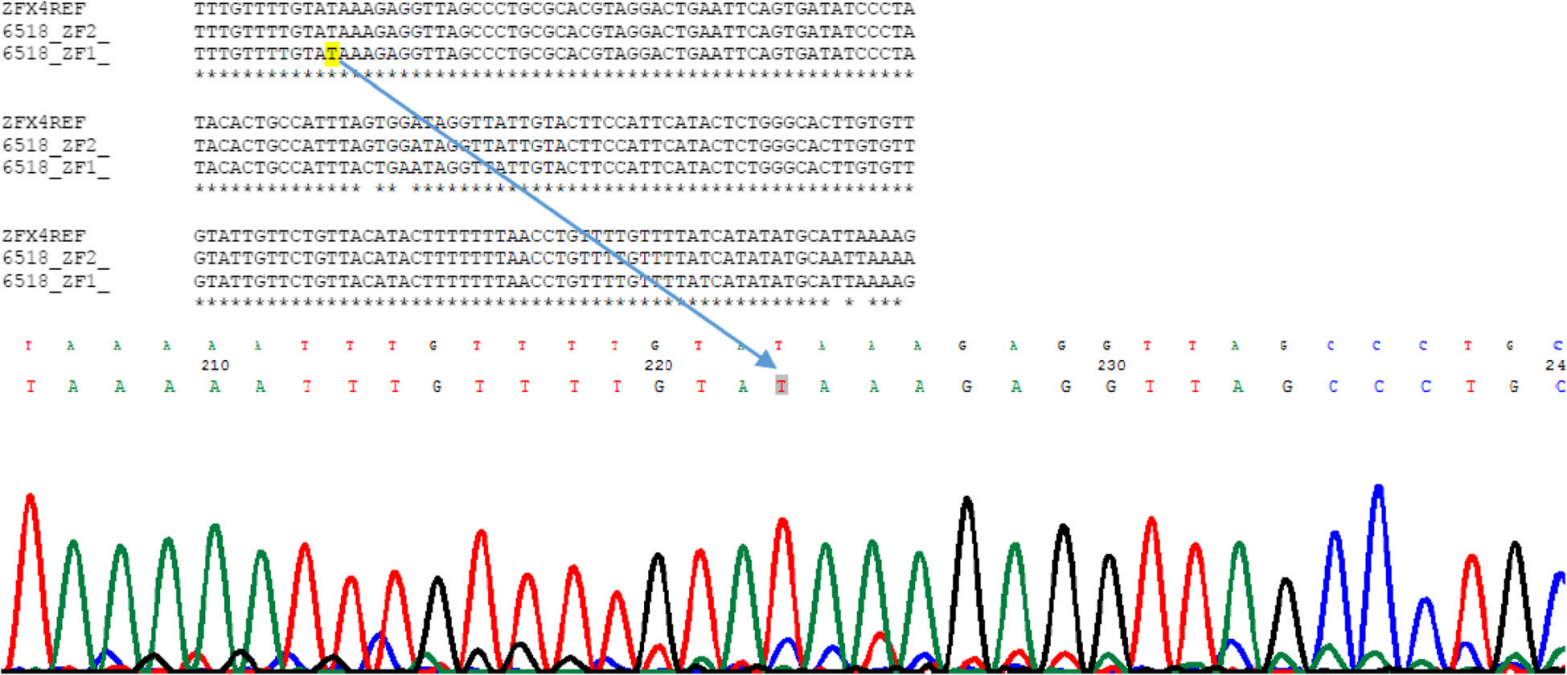
Sequence result of G12411TL4137F mutation in *ZFHX4* gene in a child with congenital Ptosis

**Fig. 4:**
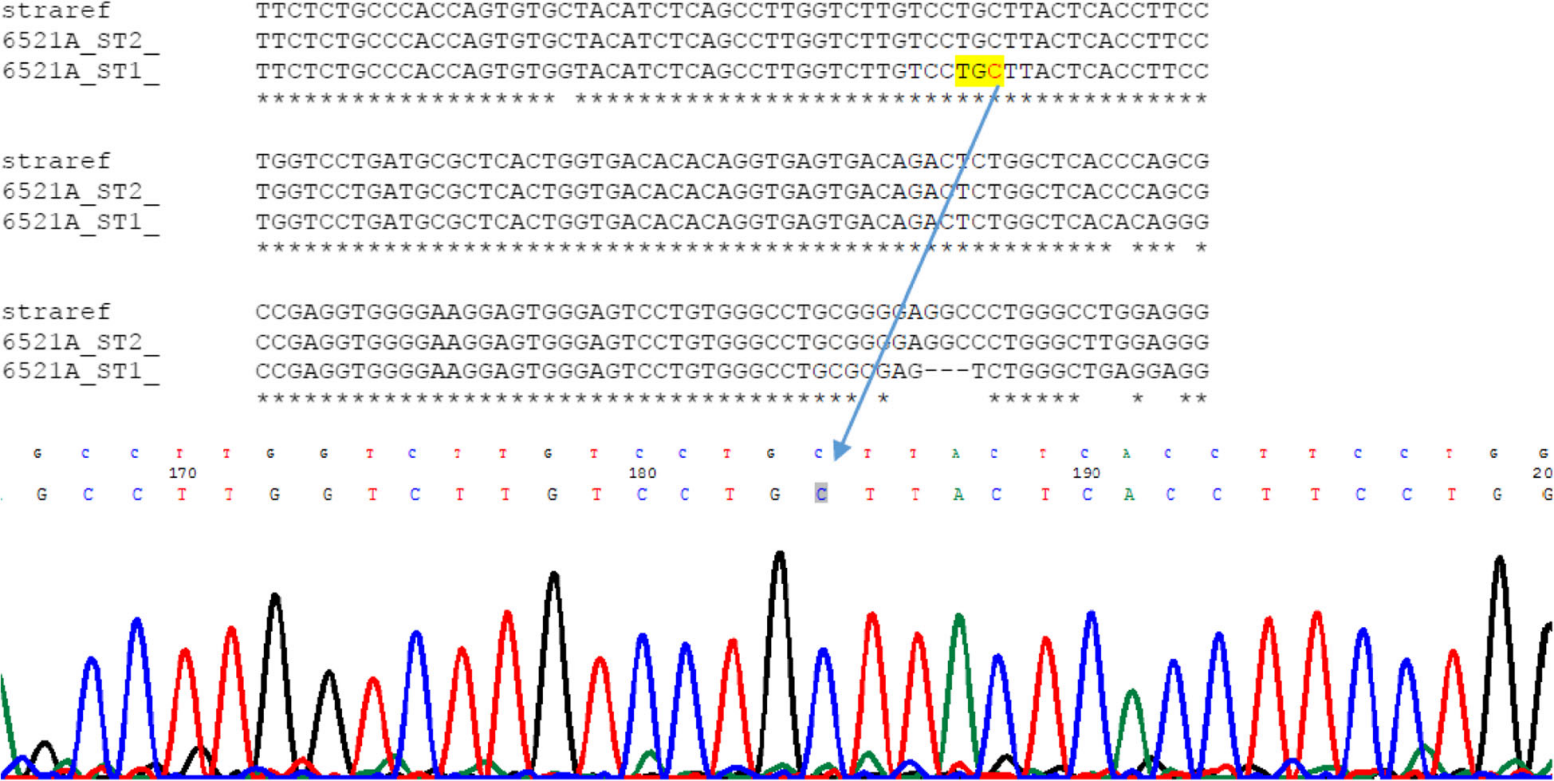
The T > C P. Y374C mutation in *STRA6* gene in a child with micropthalmous

Out of these the 30 children having congenital ocular anomalies, genetic analysis was carried out in 25 children. Five (16.7%) children were non responders. The average (SD) age of study participants was 13.3 (3.1) years. Among 25 children, fifteen children (63.3%) were male and 10 (36.7%) were female. Within this group, 17 (68%) children were from low land, 5 (20%) were from the hill region, and 3 (12%) were from the mountain region. Eleven (44%) children had congenital ptosis, 5 (20.0%) had congenital cataract, 4 (16%) had iris and chorioretinal coloboma, 3 (12%) had microphthalmos, 1 (4%) had Nevus of Ota, 1 (4%) had proptosis secondary to Crouzon syndrome, Table 1 shows the pattern of congenital ocular diseases across the three ecological regions. Most of the children with congenital anomalies were from the lowland. Out of 25 children who underwent genetic tests, meaningful genetic results could be found in 18 children. Two children were found to have mutant genes; one child with congenital ptosis and one with microphthalmous had mutation. Both of them were from lowland. The summary of different gene mutations has been shown in Table 2**.** The table shows that Ten SNP among seven genes for five congenital eye disorders listed above were selected. One missense alteration G12411T of *ZFHX4* gene was identified in patient with congenital ptosis. (Fig. 3). Another missense variation T > C P. Y374C of STRA6 gene was identified in patient with microphthalmos (Fig. 4). The proportion analysis was carried out to see the children with congenital and hereditary diseases having mutation versus non mutation in three ecological regions. (Table 3).

**Table 1:** Pattern of congenital ocular diseases in three ecological regions

Disease pattern	Low lands N(%)	Hills N(%)	Mountains N (%)
Coloboma	4 (16)	0 (0)	0 (0)
Congenital Ptosis	7 (28)	3 (12)	1 (4)
Congenital cataract	3 (12)	1 (4)	1 (4)
Microphthalmous	3 (12)	0	0
Nevus of Ota	0	0	1 (4)
Proptosis (Crouzen syndrome)	0	1 (4)	0
Total	17 (68)	5 (20)	3 (12)

**Table 2:** The result summary of genetic analysis showing gene mutation

RN	SN	Gene	Mutation	BASE Call	Result
1	A	*ZFHX4*	G12411T L4137F	T	G12411T detected
2	B	*GJA8_Cx50*	c.649G > A (Val196Met)	G	c.649G > A Not detected
3	F	*FGFR2*	S267P (T-C)	T	Not detected
3	F	*FGFR2*	C278F (G-T)	G	Not detected
3	F	*FGFR2*	Q289P (A-C)	A	Not detected
4	F	*FGFR2*	C342S (G-C)	–	Not detected
4	F	*FGFR2*	C342Y (G-A)	–	Not detected
4	F	*FGFR2*	C342W (C-G)	–	Not detected
4	F	*FGFR2*	A344A (G-A)	–	Not detected
4	F	*FGFR2*	S347C (C-G)	–	Not detected
5	D	*STRA6*	T > C P.Y374C	C	T > C P.Y374C detected
6	D	*STRA6*	A > T P.L152M	T	L152L present, mutation not detected.
7	D	*CRYBA4*	C > T P.R25W	C	Mutation not detected
8	D	*OTX2*	p. Gln104 X	C	p. Gln104 X Not detected
8	D	*OTX2*	p. Gln106 His	C	p. Gln106 His Not detected
9	D	*OTX2*	p. Thr186 Fs < frame shift	G	p. Thr186 Fs < frame shift Not detected
10	C	*ABCB6*	p. Ala 57 Thr G > A	G	p. Ala 57 Thr G > A Not detected

SN = Sample number, RN = reaction number Where, A: Congenital Ptosis, B: Congenital Cataract, C: Colobama, D: Microphthalmus, F: Crouzon Syndrome

**Table 3:** Proportion analysis of disease according to presence or absence of mutant gene and the region

Variables	category	N(%)	p
Region	Mountain	2 (8)	< 0.001
	Hill	5 (20)	
	Terai	18 (72)	
Mutation	Absent	23 (92)	< 0.001
	Present	2 (8)	

## Discussion

In the current era of genetic advances, the diagnosis of genetic eye diseases is increasing and facilitated by collaborations between ophthalmologists and geneticists. Genetic testing can not only help confirm suspected diagnoses, but also provide important prognostic information and guide management for the individual affected and help provide appropriate genetic counseling for parents [13]. A comprehensive genetic evaluation requires a thorough clinical examination, a detailed family history, and access to advanced technology for molecular investigation [14].

We carried out the genetic analysis to find out molecular pattern and any significant mutations if present in Nepalese children with inherited eye diseases. In our study, congenital anomalies were most commonly present in Lowland region. The prevalence of congenital disease being more common in plain land is may be due to the fact that ocular morbidity and blindness as a whole was more common in this region. Moreover, the practice of consanguineous marriage is common in the community of this region which might have contributed to high prevalence of hereditary ocular diseases.

In our study, five types of congenital anomalies were studied; congenital ptosis, microphthalmos, congenital cataract, coloboma and Crouzon syndrome. Among these, only crouzon syndrome was found to be familial where mother was also affected.

Hereditary cataracts are clinically and genetically heterogeneous, often presenting as congenital or developmental cataracts that arises at birth or during the first few decades of life [15]. This is one of the important causes of avoidable blindness in children. Approximately 25% of non-syndromic cataracts are inherited [16]. They can also be grouped into three major classes, based on the functions of known underlying genes, those that code for crystallins, membrane/cytoskeleton proteins, and transcription factors. At least 35 independent loci, including more than 20 known genes, have been identified for non-syndromic cataract (Cat- Map) [17, 18]. Majority of missense mutations (nearly 50%) is due to mutations in crystallin genes which is followed by mutations in the genes for cytoskeletal or membrane proteins (nearly 35%) [17]. The Gap Junction Protein Alpha 8 (*GJA8) and* Gap Junction Protein Alpha 3 (*GJA3)* mutations together account for 20% of the reported total non-syndromic familial cataracts worldwide [17]. We selected a known SNP c.649G > A (Val196Met) from the same gene *GJA8* and tested for the children with congenital cataract. This variation was not found in our patient with congenital cataract.

Another important congenital disease in our study was congenital ptosis. We selected one alteration in the ZFHX4 gene, G12411T L4137F in the children with congenital ptosis. This alteration was also reported previously in a Japanese family [19]. In our study this change was detected in one child with congenital ptosis.

Another group of children were with iris and chorioretinal coloboma and microphthalmia. Iris and Choroidal Choroidal and Microphthalmia are structurally related congenital eye malformations which display a spectrum of severity and can occur in isolation or as part of a syndrome [20]. Microphthalmia refers to a small eye, defined by axial length. Iris coloboma is a segmental ocular defect resembling a key hole deficiency in iris. Chorioretinal coloboma is associated with iris coloboma and visually significant if posterior pole is involved [21].. The STRA6 gene plays a key role in normal ocular development, encoding a transmembrane receptor for the retinol-binding protein (RBP) and is responsible for mediating vitamin A uptake from circulation to target organs including the eye [22]. We selected two SNP in STRA6 (T > C P. Y374C and A > T P. L152M) gene which are already found in database. We could identify one of these mutations in our patient.

The second gene we selected was Crystalline Beta A 4 (CRYBA4). It is a known fact that complex microphthalmia in association with genetic cataracts has been attributed to mutations in the *CRYBA4* gene [23]. We selected one SNP C > T P. R25W from CRYBA4 gene which could not be identified in our patient.

The next gene was Orthodenticle Homeobox 2 (OTX2). The *OTX2* gene encodes a transcription factor critical for forebrain and eye development [24]. The OTX2 protein contains a homeodomain, responsible for DNA binding, SGQFTP and SIWSPA motifs involved in protein–protein interactions, and two C-terminal tandem OTX-tail motifs responsible for transactivation [25]. We selected three SNP p. Gln104 X, p. Gln106 His, p. Thr186 Fs < frame shift from OTX2 gene which could not be identified in our patient.

Another gene we selected was one SNP p. Ala 57 Thr GA, from the ATP Binding Cassette Sub Family B Member 6 (ABCB6) gene, which is involved in the active transport of various compounds vital for CNS development [26]. However, we could not identify this mutation in our patient. Another important hereditary disease was Crouzon syndrome which is an autosomal dominant craniosynostosis disorder which is caused by mutation in the Fibroblast Growth Factor Receptor 2 (FGFR2) genes [27]. We selected few SNP from FGFR2 gene, for crouzon syndrome which codes for fibroblast growth factor receptor, but mutation was not found in this patient as well. The limitation of our study is the small sample size. From a large population based sample, we selected those children for genetic analysis who had congenital ocular anomalies. Sample size could have been increased by recruiting children from the hospital based data. Another limitation is the pattern of diseases which were studied. We could widen our research in other types of congenital ocular disease as well. Carrying out genetic screening in Nepal is challenging with limited resources and high cost.

## Conclusion

Our study was the first of its kind in Nepal to identify genetic mutations associated with congenital ocular anomalies. Through our analysis, two new mutations were identified in children with congenital ptosis and the microphthalmos unlike other studies on genetic analysis of congenital ptosis and microphthalmos. Further analysis of genetic factors including wide range of genetic and hereditary ocular diseases is crucial to better understand their genetic association.. This helps further in genetic counseling and probably gene therapy to prevent childhood blindness.

## Supplementary information

**Additional file 1.** Primer names with sequence and PCR products in different diseases

**Additional file 2.** Samples with further DNA sequencing. A: Congenital Ptosis B: Congenital Cataract C: Colobama D: Micropthalmus F: Crouzen Syndrome

**Additional file 3.** The results of the sequencing analysis of primers.

## Data Availability

The datasets used and/or analyzed during the current study are available in the link https://osf.io/4yezc/?view_only=cc9fbeb0693a46e4852384a606faf6ec, dbSNP reference sequences and Primer3 primer design software available at *bioinfo.ut.ee**/primer3–0.4.0.* http://www.genome.jp/tools/clustalw/

## Abbreviations

NPODS: Nepal Pediatric Ocular Disease Study)
STRA6: Signaling Receptor and Transporter Retinol 6
ZFHX4: Zinc Finger Homeobox 4
KCGRL: Kathmandu Centre for Genomics and Research Laboratory
SNP: Single Neucleotide Polymorphism
PCR: Polymerase Chain Reaction
dNTPs: Deoxy Nucleotide Triphosphates
SMQ: Standard Milli Q
SPSS: Statistical Package for Social Science
GJA8: Gap Junction Protein Alfa 8
RBP: Retinol Binding Protein
CRYBA4: Crystallin Beta A 4
OTX2: Orthodenticle Homeobox 2
ABCB6 Gene: ATP binding Cassette Subfamily B Member 6
FGFR 2: Fibroblast Growth Factor Receptor 2
