# Supplementary material for: Genetic analysis of children with congenital ocular anomalies in three ecological regions of Nepal: a phase II of Nepal pediatric ocular diseases study
Source: BMC Med Genet. 2020 Sep 22;21:185. doi: 10.1186/s12881-020-01116-9 (PMC7510079; doi:10.1186/s12881-020-01116-9)
Supplement: Supplementary file 1 — Additional file 1. Primer names with sequence and PCR products in different diseases [file 12881_2020_1116_MOESM1_ESM.docx]

Primer names with sequences and PCR products in different diseases

| **Disease Name** | **Primer Name** | **Primer Sequence** | **PCR Product** |
| --- | --- | --- | --- |
| Congenital Ptosis | ATPL_ZF2  ATPL_ZF3 | 5’TGTAGTGCAGCAACAGTTTGG 3’  5’ CATGGCAAGCAAAACAAAAAT 3’ | 445 |
| Congenital Cataract | ATPL_GJ1  ATPL_GJ2 | 5’ CTGCTGAGGACCTACATCTGC 3’  5’ CAAGGGGAAATAGTGGGAAAC 3’ | 405 |
| Crouzen Syndrome | ATPL_FG1  ATPL_FG2 | 5’ CAGCCAATAACCTGGGATGTA 3’  5’ TCCTCACCTTGAGAACCTTGA 3 | 458 |
| Crouzen Syndrome | ATPL_FG3  ATPL_FG4 | 5’ GATCAAGCACGTGGAAAAGAA 3’  5’ AGCTCTTTCCATCCACTCCAT 3’ | 448 |
| Micropthalmus | ATPL_ST1  ATPL_ST2 | 5’ AGGTGGGCTCCATGACAG 3’  5’ CATTGGCTTACACTGCATCTG 3’ | 380 |
| Micropthalmus | ATPL_ST3  ATPL_ST4 | 5’ TATTAGCCCCATTTTGCAATG 3’  5’ GAGGGTAGTAGAGGGCAGCAT 3’ | 405 |
| Micropthalmus | ATPL_CR1  ATPL_CR2 | 5 ‘GCTCCTGGAGGAATCTGAGTT 3’  5 ‘AAAAGTCCTAGCACCCGTCTC 3’ | 317 |
| Micropthalmus | ATPL_OT1  ATPL_OT2 | 5’ AGCCTCCCCAACTTTCTTACA 3’  5’ GAAGCTGGGCTCCAGATAGAC 3’ | 404 |
| Micropthalmus | ATPL_OT3  ATPL_OT4 | 5’ TGAGACCTGCCAAAAAGAAGA 3’  5’ AAGCTGGGGACTGATTGAGAT 3’ | 387 |
| Colobama | ATPL_AB1  ATPL_AB2 | 5’ CCTGAGTCCCTGCTTCTTCTT 3’  5’ AACTTGATCCAGATGCCCATT 3’ | 366 |
