# Supplementary material for: Genetic analysis of children with congenital ocular anomalies in three ecological regions of Nepal: a phase II of Nepal pediatric ocular diseases study
Source: BMC Med Genet. 2020 Sep 22;21:185. doi: 10.1186/s12881-020-01116-9 (PMC7510079; doi:10.1186/s12881-020-01116-9)
Supplement: Supplementary file 2 — Additional file 2. Samples with further DNA sequencing. A: Congenital Ptosis B: Congenital Cataract C: Colobama D: Micropthalmus F: Crouzen Syndrome [file 12881_2020_1116_MOESM2_ESM.docx]

Samples with further DNA sequencing

| **Lane Number** | **Sample Number** | **Gene** | **Mutation covered** |
| --- | --- | --- | --- |
| M | 100-1000 bp ladder | - | - |
| 1 | A | ZFHX4 | G12411T L4137F |
| 2 | B | GJA8_Cx50 | c.649G>A (Val196Met) |
| 3 | F | FGFR2 | S267P (T-C) |
| 3 | F | FGFR2 | C278F (G-T) |
| 3 | F | FGFR2 | Q289P (A-C) |
| 4 | F | FGFR2 | C342S (G-C) |
| 4 | F | FGFR2 | C342Y (G-A) |
| 4 | F | FGFR2 | C342W (C-G) |
| 4 | F | FGFR2 | A344A (G-A) |
| 4 | F | FGFR2 | S347C (C-G) |
| 5 | D | STRA6 | T>C P.Y374C |
| 6 | D | STRA6 | A>T P.L152M |
| 7 | D | CRYBA4 | C>T P.R25W |
| 8 | D | OTX2 | p. Gln104 X |
| 8 | D | OTX2 | p. Gln106 His |
| 9 | D | OTX2 | p. Thr186 Fs<frame shift |
| 10 | C | ABCB6 | p. Ala 57 Thr G^^A |

A: Congenital Ptosis

B: Congenital Cataract

C: Colobama

D: Micropthalmus

F: Crouzen Syndrome
