# Supplementary material for: Genetic analysis of children with congenital ocular anomalies in three ecological regions of Nepal: a phase II of Nepal pediatric ocular diseases study
Source: BMC Med Genet. 2020 Sep 22;21:185. doi: 10.1186/s12881-020-01116-9 (PMC7510079; doi:10.1186/s12881-020-01116-9)
Supplement: Supplementary file 3 — Additional file 3. The results of the sequencing analysis of primers. [file 12881_2020_1116_MOESM3_ESM.doc]

**DNA sequencing**

Cycle sequencing reaction for each sample was performed in 0.2mL PCR tube. The reaction included Terminator Ready Reaction Mix (BigDye® Terminator v3.1 Cycle Sequencing Kit, Applied Biosystems) (0.5 µL), BigDye® Sequencing Buffer (1.8 µL), One sequencing primer (3.2 pmol), and template DNA (2 µL) with standard milliQ (SMQ) water (4.7 µL) to make up the volume of 10.0µL. For each sample two sequencing reactions were performed one using forward primer and other with reverse primer. The cycle sequencing protocol was as follows: Initial denaturation at 96 ºC for 1 min, followed by 25 cycles of 96ºC for 10 sec, annealing at 50 ºC for 5 sec, and elongation at 60 ºC for 4 minutes. Rapid thermal ramp to 4ºC and hold until ready to purify.

Sequencing clean up protocol: Excess dye terminators in sequencing reactions obscure data in the early part of the sequence and can interfere with base calling. Hence, best results are obtained when unincorporated dye terminators are completely removed prior to electrophoresis. As recommended by Applied Biosystems, ethanol/EDTA precipitation as a clean-up protocol. 125mM EDTA (5 µL) and Absolute ethanol (60 µL) was added to tubes and incubated at room temperature for 15 min and centrifuged at 4ºC,12000 rpm for 20 min. Supernatant was discarded

and pellet was given two successive washes in 70% ethanol (60 µL) (centrifuged at 4ºC, 10000 rpm for 10 min). The sample was then loaded on a 96 well plate (Applied Biosystems, USA) and capillary electrophoresis was performed in 3130 Genetic Analyzer (Applied Biosystems, Life Technologies). Sequence raw data generated was analyzed using SequencingAnalysis5.2 Software.

**Sequence analysis**

Analyzed sequence data files were recovered from sequencing machine in .ab1 file format. These sequences were exported in FASTA format in a word document. Sequence data for forward primer was used as it is. Sequence from reverse primer was initially reverse complemented using ChromasPro version 1.34 and then exported to word document. These two sequences were aligned with reference sequence for each mutation location using Clustal W multiple sequence alignment tool available online at <http://www.genome.jp/tools/clustalw/>Alignment used default scoring matrix IUB for DNA sequences. Through alignment it was evident the sequences belong to the respective gene as it showed higher percentage of alignment score. The location of point mutation was scanned manually to find out whether the sample has wild type nucleotide base or it harbors mutation. The sequence data for that location was simultaneously scanned in .ab1 files to confirm that it shows same peak profile. Such confirmed results were recorded in final mutation scoring table.

| **Supplementary Analysis** | | | |  |  |  |  |  |  |
| --- | --- | --- | --- | --- | --- | --- | --- | --- | --- |
|  |  |  |  |  |  |  |  |  |  |
| **SAMPLE A** |  | **ZFHX4** | |  |  | G12411T L4137F) |  |  |  |
|  |  |  |  |  |  |  |

>ZFX4REF

CAAGGTACTTGCTTGTGTCTGAACTCTAGTGCACTTATGATTTTGTAGACCATGTGAAATTTAATAAGATACCTTTTTTTTCCTTTCTTTGTGTGTAGTGCAGCAACAGTTTGGTCTGCATTTGTTAGA AGTTTAACTCCTAACAACCCAAAGACCTATTTAACAATTGGTGCATAAATGAAAGTAGTACTGTATACTTGAAACTGTTTAAGTACAAGTTGAACAAAAATTATGAAAAGGTATATTTGCTTCTCGGGA AAGCAAAGAAGCTGCTTTAAAAAATAAAAAGGGGACTAAAAATTTGTTTTGTATAAAGAGGTTAGCCCTGCGCACGTAGGACTGAATTCAGTGATATCCCTATACACTGCCATTTAGTGGATAGGTTAT TGTACTTCCATTCATACTCTGGGCACTTGTGTTGTATTGTTCTGTTACATACTTTTTTTAACCTGTTTTGTTTTATCATATATGCATTAAAAGTATTATCTTTATCAACATTTGCTGCTACTGTGTTAA CATTTTTGTTTTGCTTGCCATGAATTTCAACTTCCACCACCCAGTGAATTGATTTATAAATTGCTATGCTTTGCTGTTTTTCTGT

>6518(ZF1)

CTTTGTAAAAAGTTAACTCCTAACACCCAAAGACCTATTTAACAATTGGTGCATAAATGAAAGTAGTACTGTATACTTGAAACTGTTTAAGTACAAGTTGAACAAAAATTATGAAAAGGTATATTTGCT TCTCGGGAAAGCAAAGAAGCTGCTTTAAAAAATAAAAAGGGGACTAAAAATTTGTTTTGTATAAAGAGGTTAGCCCTGCGCACGTAGGACTGAATTCAGTGATATCCCTATACACTGCCATTTACTGAA TAGGTTATTGTACTTCCATTCATACTCTGGGCACTTGTGTTGTATTGTTCTGTTACATACTTTTTTTAACCTGTTTTGTTTTATCATATATGCATTAAAAGTATTATCTTTATCAACATTTGCTGCTAC TGTGTTAACATTTTTGTTTTGCTTGCCATAA

>6518(ZF2)

TTTCTGTAGTGCAGCAACAGTTTGGTCTGCATTTGTTAGAAGTTTAACTCCTAACAACCCAAAGACCTATTTAACAATTGGTGCATAAATGAAAGTAGTACTGTATACTTGAAACTGTTTAAGTACAAG

TTGAACAAAAATTATGAAAAGGTATATTTGCTTCTCGGGAAAGCAAAGAAGCTGCTTTAAAAAATAAAAAGGGGACTAAAAATTTGTTTTGTATAAAGAGGTTAGCCCTGCGCACGTAGGACTGAATTC

AGTGATATCCCTATACACTGCCATTTAGTGGATAGGTTATTGTACTTCCATTCATACTCTGGGCACTTGTGTTGTATTGTTCTGTTACATACTTTTTTTAACCTGTTTTGTTTTATCATATATGCAATT

AAAAAATGGTA

| ZFX4REF | CAAGGTACTTGCTTGTGTCTGAACTCTAGTGCACTTATGATTTTGTAGACCATGTGAAAT | | |
| --- | --- | --- | --- |
| 6518_ZF2_ | ------------------------------------------------------------ | |  |
| 6518_ZF1_ | ------------------------------------------------------------ | |  |
| ZFX4REF | TTAATAAGATACCTTTTTTTTCCTTTCTTTGTGTGTAGTGCAGCAACAGTTTGGTCTGCA | | |
| 6518_ZF2_ | ----------------------------- | | TTTCTGTAGTGCAGCAACAGTTTGGTCTGCA |
| 6518_ZF1_ | ----------------------------------------------------------- | | C |
| ZFX4REF | TTTGTTAGAAGTTTAACTCCTAACAACCCAAAGACCTATTTAACAATTGGTGCATAAATG | | |
| 6518_ZF2_ | TTTGTTAGAAGTTTAACTCCTAACAACCCAAAGACCTATTTAACAATTGGTGCATAAATG | | |
| 6518_ZF1_ | TTTGTAAAAAGTT-AACTCCTAACA-CCCAAAGACCTATTTAACAATTGGTGCATAAATG | | |
|  | ***** * ***** *********** ********************************** | | |
| ZFX4REF | AAAGTAGTACTGTATACTTGAAACTGTTTAAGTACAAGTTGAACAAAAATTATGAAAAGG | | |
| 6518_ZF2_ | AAAGTAGTACTGTATACTTGAAACTGTTTAAGTACAAGTTGAACAAAAATTATGAAAAGG | | |
| 6518_ZF1_ | AAAGTAGTACTGTATACTTGAAACTGTTTAAGTACAAGTTGAACAAAAATTATGAAAAGG | | |
|  | ************************************************************ | | |
| ZFX4REF | TATATTTGCTTCTCGGGAAAGCAAAGAAGCTGCTTTAAAAAATAAAAAGGGGACTAAAAA | | |
| 6518_ZF2_ | TATATTTGCTTCTCGGGAAAGCAAAGAAGCTGCTTTAAAAAATAAAAAGGGGACTAAAAA | | |
| 6518_ZF1_ | TATATTTGCTTCTCGGGAAAGCAAAGAAGCTGCTTTAAAAAATAAAAAGGGGACTAAAAA | | |
|  | ************************************************************ | | |
| ZFX4REF | TTTGTTTTGTATAAAGAGGTTAGCCCTGCGCACGTAGGACTGAATTCAGTGATATCCCTA | | |
| 6518_ZF2_ | TTTGTTTTGTATAAAGAGGTTAGCCCTGCGCACGTAGGACTGAATTCAGTGATATCCCTA | | |
| 6518_ZF1_ |  |  | TTTGTTTTGTATAAAGAGGTTAGCCCTGCGCACGTAGGACTGAATTCAGTGATATCCCTA |
|  | ************************************************************ | | |
| ZFX4REF | TACACTGCCATTTAGTGGATAGGTTATTGTACTTCCATTCATACTCTGGGCACTTGTGTT | | |
| 6518_ZF2_ | TACACTGCCATTTAGTGGATAGGTTATTGTACTTCCATTCATACTCTGGGCACTTGTGTT | | |
| 6518_ZF1_ | TACACTGCCATTTACTGAATAGGTTATTGTACTTCCATTCATACTCTGGGCACTTGTGTT | | |
|  | ************** ** ****************************************** | | |
| ZFX4REF | GTATTGTTCTGTTACATACTTTTTTTAACCTGTTTTGTTTTATCATATATGCATTAAAAG | | |
| 6518_ZF2_ | GTATTGTTCTGTTACATACTTTTTTTAACCTGTTTTGTTTTATCATATATGCAATTAAAA | | |
| 6518_ZF1_ | GTATTGTTCTGTTACATACTTTTTTTAACCTGTTTTGTTTTATCATATATGCATTAAAAG | | |
|  | ***************************************************** * *** | | |


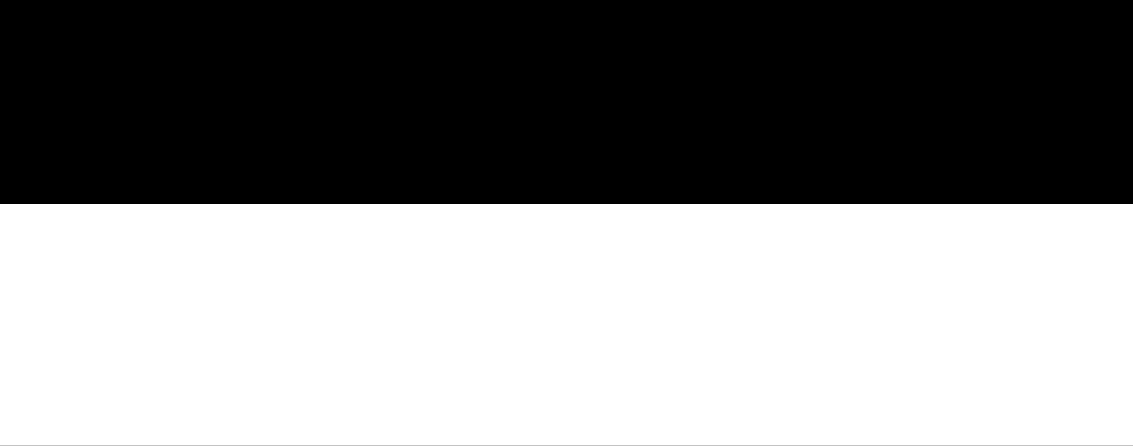

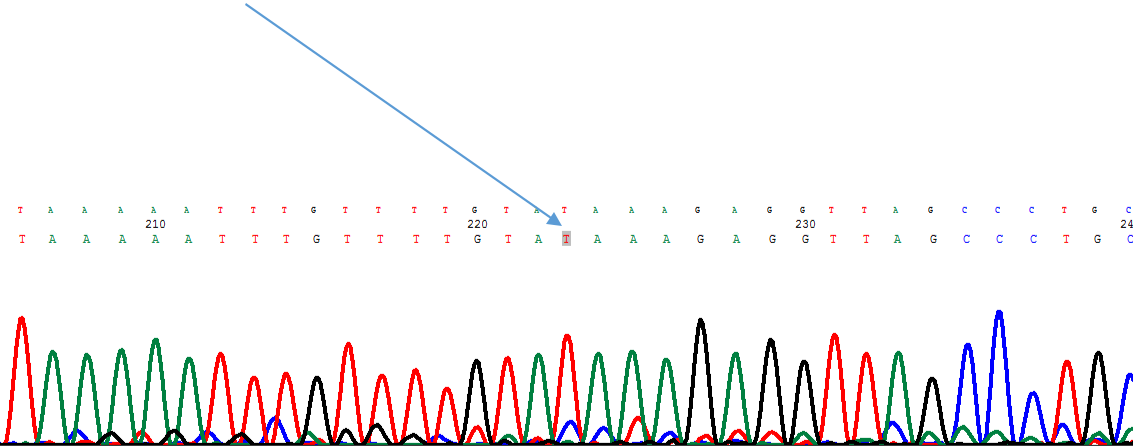


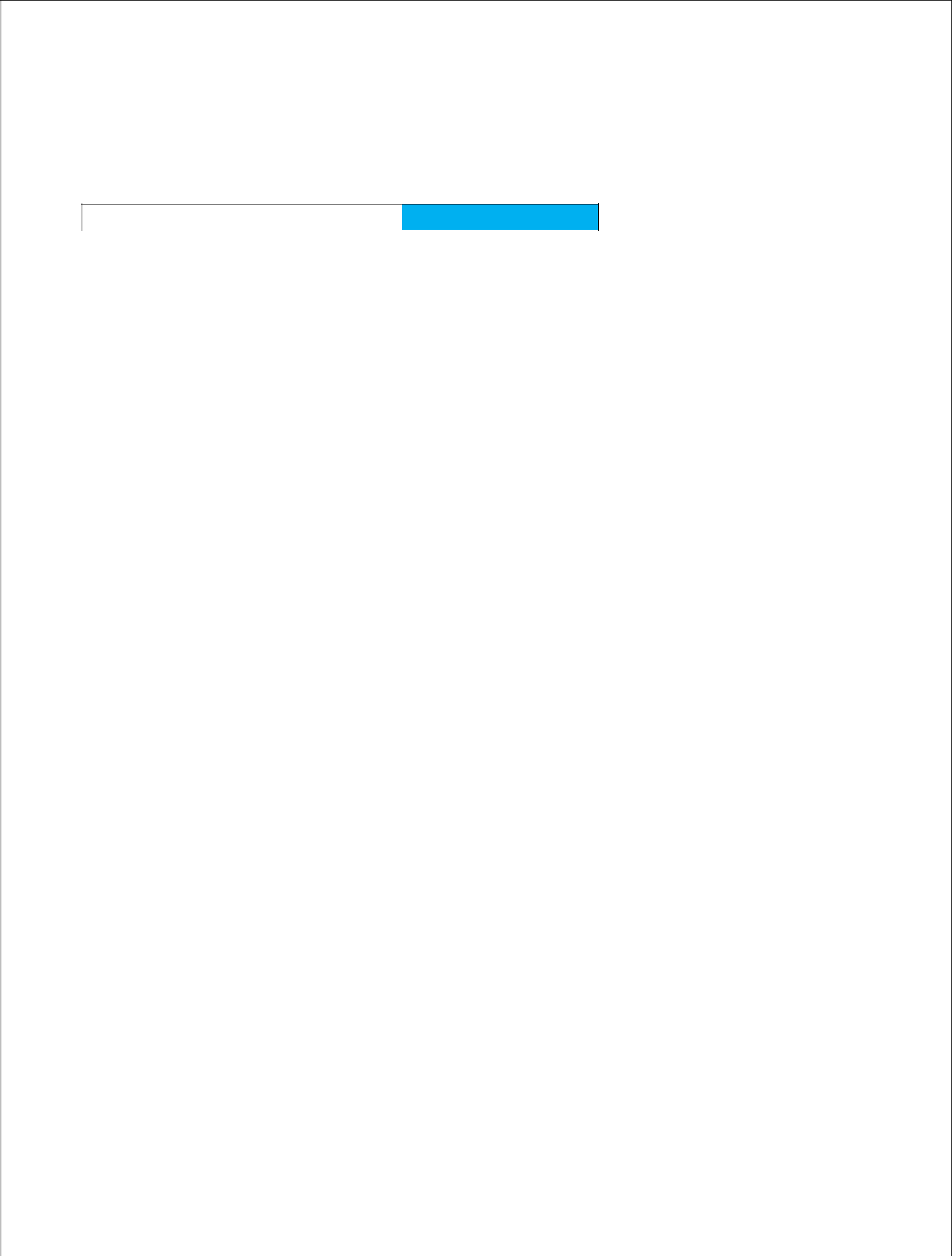


| **Sample B** | **GJA8/Cx50** | c.649G>A (Val196Met) |
| --- | --- | --- |
|  |

Nucleotide change GTG^🡪^ATG (Val to Met)

>GJA8_Cx50 c.649G>A (Val196Met)

>GJA8_Cx50

CGGCGGCCCGGACCAGGGCAGCGTCAAGAAGAGCAGCGGCAGCAAAGGCACTAAGAAGTTCCGGCTGGAGGGGACCCTGCTGAGGACCTACATCTGC

CACATCATCTTCAAGACCCTCTTTGAAGTGGGCTTCATCGTGGGCCACTACTTCCTGTACGGGTTCCGGATCCTGCCTCTGTACCGCTGCAGCCGGT

GGCCCTGCCCCAATGTGGTGGACTGCTTCGTG**TCC**CGGCCCACGGAGAAAACCATCTTCATCCTGTTCATGTTGTCTGTGGCCTCTGTGTCCCTATT

CCTCAACGTGATGGAGTTGGGCCACCTGGGCCTGAAGGGGATCCGGTCTGCCTTGAAGAGGCCTGTAGAGCAGCCCCTGGGGGAGATTCCTGAGAAA

TCCCTCCACTCCATTGCTGTCTCCTCCATCCAGAAAGCCAAGGGCTATCAGCTCCTAGAAGAAGAGAAAATCGTTTCCCACTATTTCCCCTTGACCG

AGGTTGGGATGGTGGA

>6519(GJ1)

CTTACAACCTCCCCTTTTTAAAGTGGGCTTCATCGTGGGCCACTACTTCCTGTACGGGTTCCGGATCCTGCCTCTGTACCGCTGCAGCCGGTGGCCC

TGCCCCAATGTGGTGGACTGCTTCGTGTCCCGGCCCACGGAGAAAACCATCTTCATCCTGTTCATGTTGTCTGTGGCCTCTGTGTCCCTATTCCTCA

ACGTGATGGAGTTGGGCCACCTGGGCCTGAAGGGGATCCGGTCTGCCTTGAAGAGGCCTGTAGAGCAGCCCCTGGGGGAGATTCCTGAGAAATCCCT

CCACTCCATTGCTGTCTCCTCCATCCAGAAAGCCAAGGGCTATCAGCTTCTAGAAAAAGAGAAAATCGTTTCCCACTATTTCCCCTTAAAA

>6519(GJ2)

CTGCTGAGGACCTACATCTGCCACATCATCTTCAAGACCCTCTTTGAAGTGGGCTTCATCGTGGGCCACTACTTCCTGTACGGGTTCCGGATCCTGC CTCTGTACCGCTGCAGCCGGTGGCCCTGCCCCAATGTGGTGGACTGCTTCGTGTCCCGGCCCACGGAGAAAACCATCTTCATCCTGTTCATGTTGTC TGTGGCCTCTGTGTCCCTATTCCTCAACGTGATGGAGTTGGGCCACCTGGGCCTGAAGGGGATCCGGTCTGCCTTGAAGAGGCCTGTAGAGCAGCCC CTGGGGGAGATTCCTGAGAAATCCCTCCACTCCATTGCTGTCTCCTCCATCCAGAAAGCCAAGGGCTATCATGACGATGATGTCCCTCGTCG

| GJA8_Cx50 | CCGGCTGGAGGGGACCCTGCTGAGGACCTACATCTGCCACATCATCTTCAAGACCCTCTT | | | |
| --- | --- | --- | --- | --- |
| 6519_GJ1_ | -------------------------------------CTTACAACCTCC----- | | | CCTTTT |
| 6519_GJ2_ | ----------------CTGCTGAGGACCTACATCTGCCACATCATCTTCAAGACCCTCTT | | | |
|  | * * **** | | | *** ** |
| GJA8_Cx50 | TGAAGTGGGCTTCATCGTGGGCCACTACTTCCTGTACGGGTTCCGGATCCTGCCTCTGTA | | | |
| 6519_GJ1_ | TAAAGTGGGCTTCATCGTGGGCCACTACTTCCTGTACGGGTTCCGGATCCTGCCTCTGTA | | | |
| 6519_GJ2_ | TGAAGTGGGCTTCATCGTGGGCCACTACTTCCTGTACGGGTTCCGGATCCTGCCTCTGTA | | | |
|  | * ********************************************************** | | | |
| GJA8_Cx50 | CCGCTGCAGCCGGTGGCCCTGCCCCAATGTGGTGGACTGCTTCGTGTCCCGGCCCACGGA | | | |
| 6519_GJ1_ | CCGCTGCAGCCGGTGGCCCTGCCCCAATGTGGTGGACTGCTTCGTGTCCCGGCCCACGGA | | | |
| 6519_GJ2_ | CCGCTGCAGCCGGTGGCCCTGCCCCAATGTGGTGGACTGCTTCGTG |  | TCCCGGCCCACGGA | |
|  | ************************************************************ | | | |
| GJA8_Cx50 | GAAAACCATCTTCATCCTGTTCATGTTGTCTGTGGCCTCTGTGTCCCTATTCCTCAACGT | | | |
| 6519_GJ1_ | GAAAACCATCTTCATCCTGTTCATGTTGTCTGTGGCCTCTGTGTCCCTATTCCTCAACGT | | | |
| 6519_GJ2_ | GAAAACCATCTTCATCCTGTTCATGTTGTCTGTGGCCTCTGTGTCCCTATTCCTCAACGT | | | |
|  | ************************************************************ | | | |
| GJA8_Cx50 | GATGGAGTTGGGCCACCTGGGCCTGAAGGGGATCCGGTCTGCCTTGAAGAGGCCTGTAGA | | | |
| 6519_GJ1_ | GATGGAGTTGGGCCACCTGGGCCTGAAGGGGATCCGGTCTGCCTTGAAGAGGCCTGTAGA | | | |
| 6519_GJ2_ | GATGGAGTTGGGCCACCTGGGCCTGAAGGGGATCCGGTCTGCCTTGAAGAGGCCTGTAGA | | | |
|  | ************************************************************ | | | |
| GJA8_Cx50 | GCAGCCCCTGGGGGAGATTCCTGAGAAATCCCTCCACTCCATTGCTGTCTCCTCCATCCA | | | |
| 6519_GJ1_ | GCAGCCCCTGGGGGAGATTCCTGAGAAATCCCTCCACTCCATTGCTGTCTCCTCCATCCA | | | |
| 6519_GJ2_ | GCAGCCCCTGGGGGAGATTCCTGAGAAATCCCTCCACTCCATTGCTGTCTCCTCCATCCA | | | |
|  | ************************************************************ | | | |


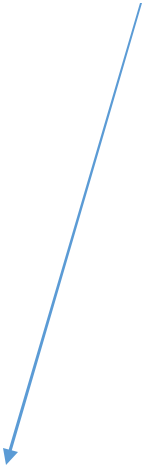


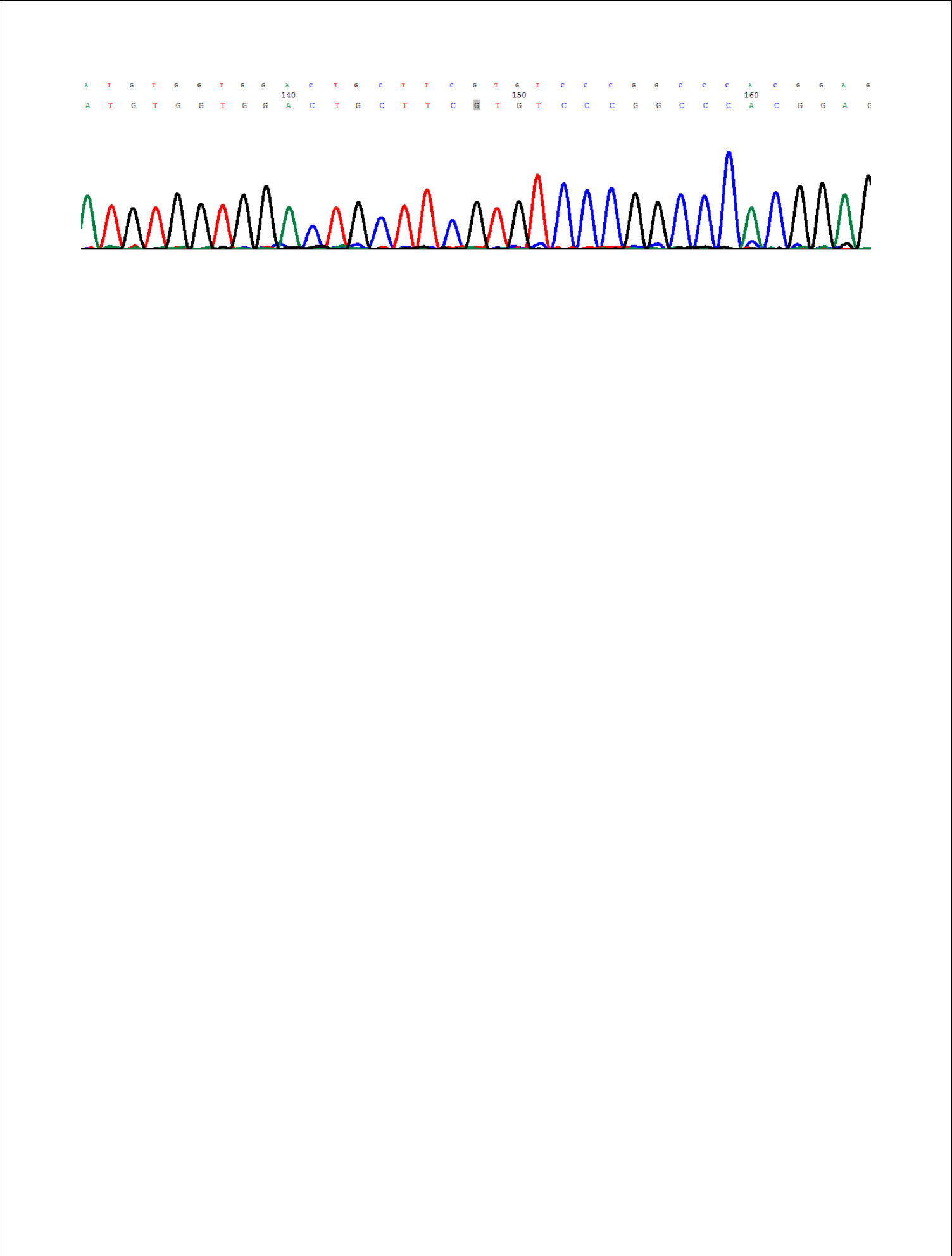


| **Sample F** | **FGFR2** |  | S267P (T-C) |  |
| --- | --- | --- | --- | --- |
|  |  |  |
|  |  |  | C278F (G-T) |  |
|  |  |  | Q289P (A-C) |  |

>FGFR2 C278F (G-T)

TTCTTCCCGTATTCATCAGGTGGCAGTGGACAGCCAATAACCTGGGATGTAATAAGTTCTTTTCTCAATTTTCTAAGTAAGTGTTCTTTTTACAAGG GTCGCGCTCCGGCAGTCTCCTTTGAAGTCGTTTCTGTTATTCATGGGGCCACAGTGTTATTTCAAAGGTGTCAGCCAGCAGGCTTGAGGCTTTTCTG GCATGAGGTCACTGACAGCCCTCTGGACAACACAGCTTATTTATTGGTCTCTCATTCTCCCATCCCCACTCCTCCTTTCTTCCCTCTCTCCACCAGA GCGATCGCCTCACCGGCCCATCCTCCAAGCCGGACTGCCGGCAAATGCCTCCACAGTGGTCGGAGGAGACGTAGAGTTTGTCTGCAAGGTTTACAGT GATGCCCAGCCCCACATCCAGTGGATCAAGCACGTGGAAAAGAACGGCAGTAAATACGGGCCCGACGGGCTGCCCTACCTCAAGGTTCTCAAGGTGA GGACTTTCTGAATCTAAAGGTACCCACAACTGGGGTCTCCTTCATGGGTTTGGCCACAGGTTCTTTGATTTCCTGTTGGAGTTGAGAGAGGATGATT CTCTTTTTTGACTAGCCAGCAGAGAGTGTTCTAAGGAATTAACAGATCATTACACTTGCTAGTAGAATTTCAGAAGGGAACTATGGAGTAGGGGAAG AACTACTAAACTTGGGGAAGAACTACTAAACTTGGAGAGAGAATAGTTCAGCTATTTATCAGCCCTGAGATCGCAGACATTTAGGCTTAGCTGCGCC TCTATAAAAGTAGAGATCGTGATACTCTGTCCCCCATAGGGCGGTTGTGCAGAATAAATGGGATGGAGTGGATGGAAAGAGCTTTGTAGGCTCAAGG CATTGTGCCAGTGTTGATTGTTACTCTGATGTTGTTTTCTATTAATAGGACATTAGGATCCAATTTTAGTAGCCACGTTTTAGAAACAATTTGGATT TTTTTTTTAACAAAAACAAAACAAAACAAAA

>6520A(FG1)

ATTCCCCAACTTTTCAAAGTAAGTGTTCTTTTTACAAGGGTCGCGCTCCAGCAGTCTCCTTTGAAGTCGTTTCTGTTATTCATGGGGCCACAGTGTT ATTTCAAAGGTGTCAGCCAGCAGGCTTGAGGCTTTTCTGGCATGAGGTCACTGACAGCCCTCTGGACAACACAACTTATTTATTGGTCTCTCATTCT CCCATCCCCACTCCTCCTTTCTTCCCTCTCTCCACCAGAGCGATCGCCTCACCGGCCCATCCTCCAAGCCGGACTGCCGGCAAATGCCTCCACAGTG GTCGGAGGAGACGTAGAGTTTGTCTGCAAGGTTTACAGTGATGCCCAGCCCCACATCCAGTGGATCAAGCACGTGGAAAAGAACGGCAGTAAATACG GGCCCGACGGGCTGCCCTACCTCAAGGTTCTAAGGGTGAGGAAA

>6520A(FG2)

TTAGCCAATAACCTGGGATGTAATAAGTTCTTTTCTCAATTTTCTAAGTAAGTGTTCTTTTTACAAGGGTCGCGCTCCAGCAGTCTCCTTTGAAGTC GTTTCTGTTATTCATGGGGCCACAGTGTTATTTCAAAGGTGTCAGCCAGCAGGCTTGAGGCTTTTCTGGCATGAGGTCACTGACAGCCCTCTGGACA ACACAACTTATTTATTGGTCTCTCATTCTCCCATCCCCACTCCTCCTTTCTTCCCTCTCTCCACCAGAGCGATCGCCTCACCGGCCCATCCTCCAAG CCGGACTGCCGGCAAATGCCTCCACAGTGGTCGGAGGAGACGTAGAGTTTGTCTGCAAGGTTTACAGTGATGCCCAGCCCCACATCCAGTGGATCAA GCACGTGGAAAAGAACGGCCCGTAAATCCCGGCCCCCACCGCGCGC

FGFR2 TTTCTCAATTTTCTAAGTAAGTGTTCTTTTTACAAGGGTCGCGCTCCGGCAGTCTCCTTT

6520A_FG1_ TCCCCAACTTTTCAAAGTAAGTGTTCTTTTTACAAGGGTCGCGCTCCAGCAGTCTCCTTT

6520A_FG2_ TTTCTCAATTTTCTAAGTAAGTGTTCTTTTTACAAGGGTCGCGCTCCAGCAGTCTCCTTT

- * * ***** ********************************* ************

FGFR2 GAAGTCGTTTCTGTTATTCATGGGGCCACAGTGTTATTTCAAAGGTGTCAGCCAGCAGGC

6520A_FG1_ GAAGTCGTTTCTGTTATTCATGGGGCCACAGTGTTATTTCAAAGGTGTCAGCCAGCAGGC

6520A_FG2_ GAAGTCGTTTCTGTTATTCATGGGGCCACAGTGTTATTTCAAAGGTGTCAGCCAGCAGGC

************************************************************

FGFR2 TTGAGGCTTTTCTGGCATGAGGTCACTGACAGCCCTCTGGACAACACAGCTTATTTATTG

6520A_FG1_ TTGAGGCTTTTCTGGCATGAGGTCACTGACAGCCCTCTGGACAACACAACTTATTTATTG

6520A_FG2_ TTGAGGCTTTTCTGGCATGAGGTCACTGACAGCCCTCTGGACAACACAACTTATTTATTG

************************************************ ***********

FGFR2 GTCTCTCATTCTCCCATCCCCACTCCTCCTTTCTTCCCTCTCTCCACCAGAGCGATCGCC

6520A_FG1_ GTCTCTCATTCTCCCATCCCCACTCCTCCTTTCTTCCCTCTCTCCACCAGAGCGATCGCC

6520A_FG2_ GTCTCTCATTCTCCCATCCCCACTCCTCCTTTCTTCCCTCTCTCCACCAGAGCGATCGCC

************************************************************

| FGFR2 | TCACCGGCCCATCCTCCAAGCCGGACTGCCGGCAAATGCCTCCACAGTGGTCGGAGGAGA | | | | | | |
| --- | --- | --- | --- | --- | --- | --- | --- |
| 6520A_FG1_ | TCACCGGCCCATCCTCCAAGCCGGACTGCCGGCAAATGCCTCCACAGTGGTCGGAGGAGA | | | | | | |
| 6520A_FG2_ | TCACCGGCCCATCCTCCAAGCCGGACTGCCGGCAAATGCC | | | TCC | ACAGTGGTCGGAGGAGA | | |
|  | ************************************************************ | | | | | | |
| FGFR2 | CGTAGAGTTTGTCTGCAAGGTTTACAGTGATGCCCAGCCCCACATCCAGTGGATCAAGCA | | | | | | |
| 6520A_FG1_ | CGTAGAGTTTGTCTGCAAGGTTTACAGTGATGCCCAGCCCCACATCCAGTGGATCAAGCA | | | | | | |
| 6520A_FG2_ | CGTAGAGTTTGTC | TGC | AAGGTTTACAGTGATGCCCAGCCCCACATC | | | CAG | TGGATCAAGCA |
|  | ************************************************************ | | | | | | |
| FGFR2 | CGTGGAAAAGAACGGCA-GTAAATACGGGCCCG-ACGGGCTGCCCTACCTCAAGGTTCTC | | | | | | |
| 6520A_FG1_ | CGTGGAAAAGAACGGCA-GTAAATACGGGCCCG-ACGGGCTGCCCTACCTCAAGGTTCTA | | | | | | |
| 6520A_FG2_ | CGTGGAAAAGAACGGCCCGTAAATCCCGGCCCCCACCGCGCGC----------------- | | | | | | |
|  | **************** ****** * ***** ** * ** | | | |  |  |  |


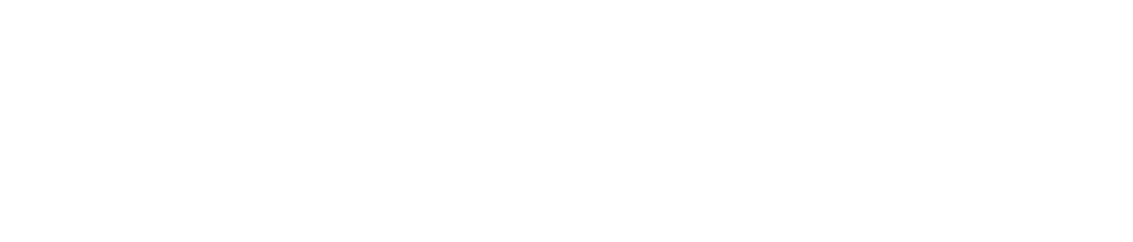

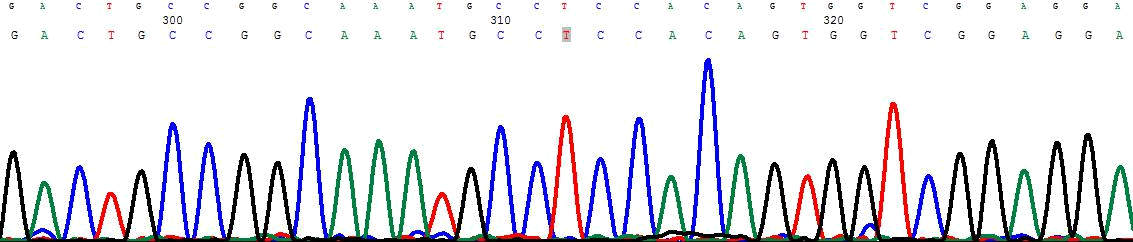

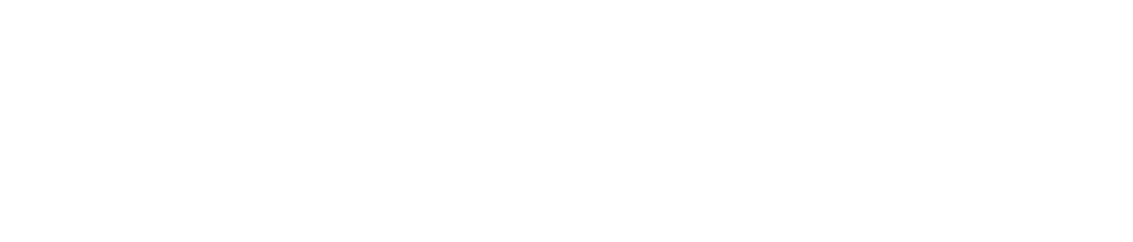

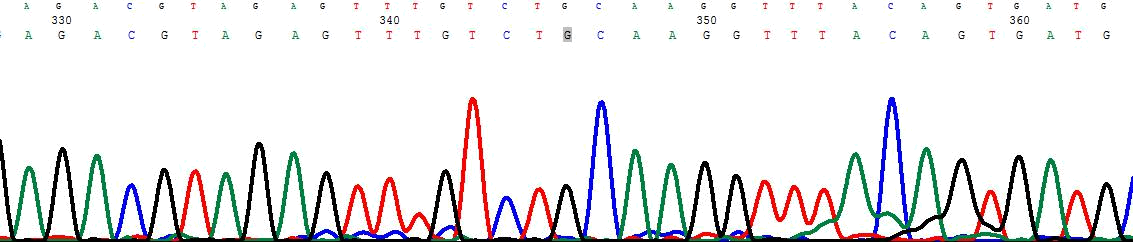

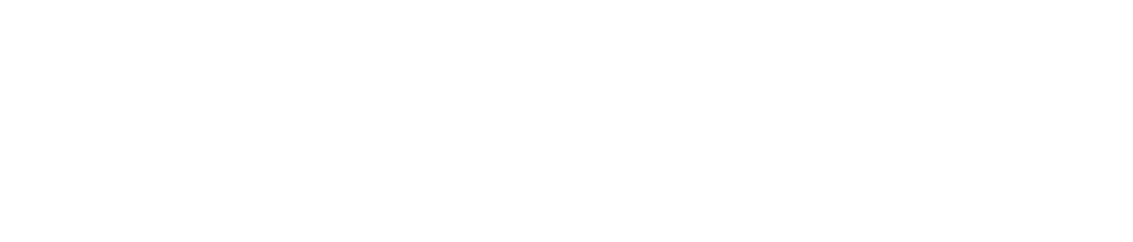

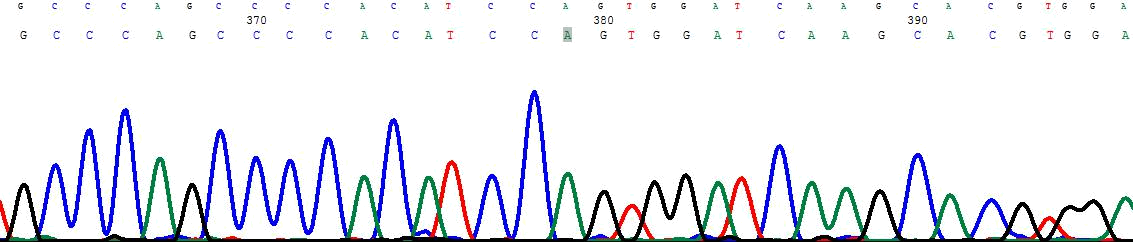


| 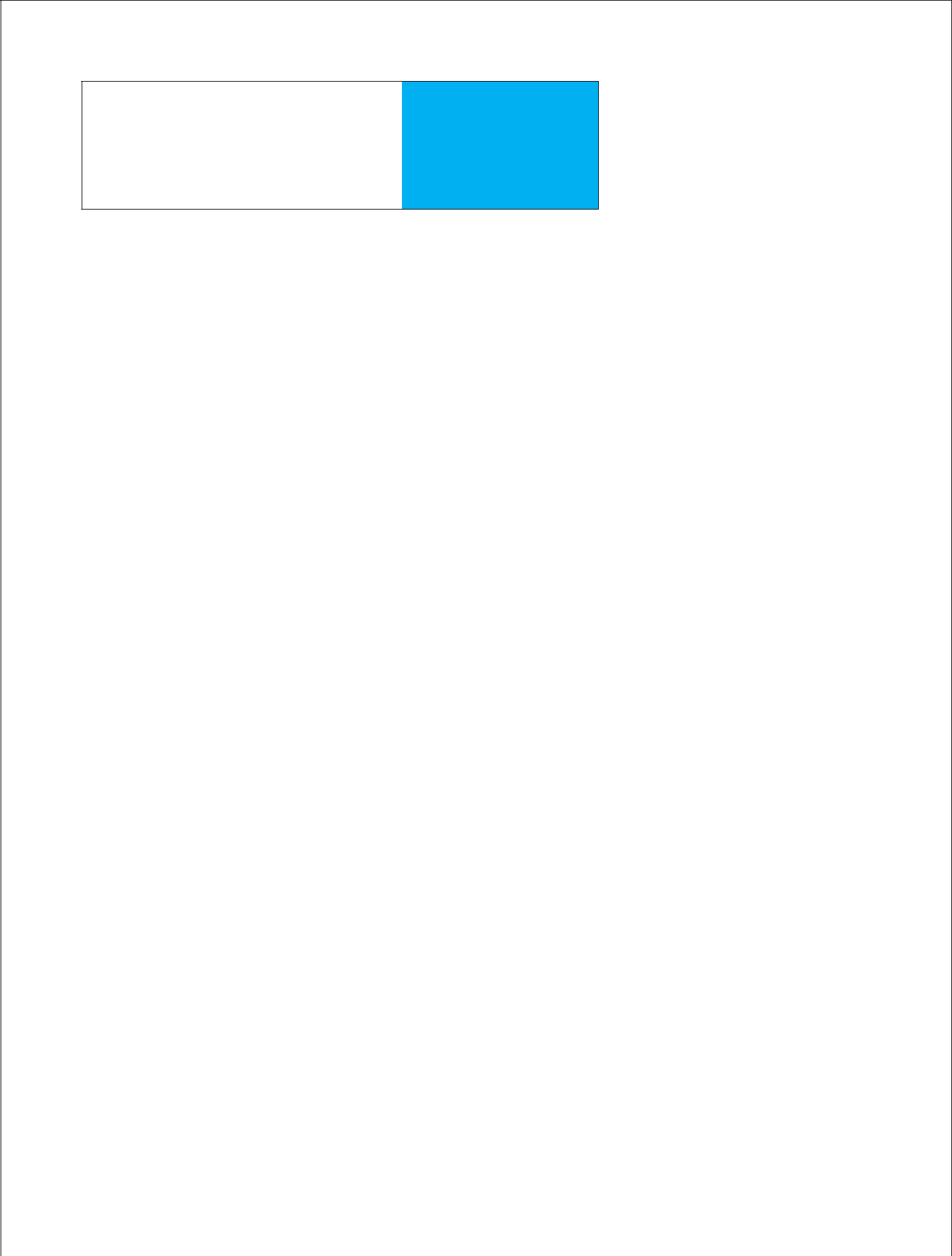Sample F | FGFR2 |  |
| --- | --- | --- |
|  |  | C342S (G-C) |
|  |  |  |
| **ANALYSIS USING** | |  |
|  |  | C342Y (G-A) |
|  |  |  |
| **NEW PRIMER SET** | | C342W (C-G) |
|  |  |  |
|  |  | A344A (G-A) |

S347C (C-G)

>FGFR2 C342Y (G-A) rs121918487 (p.Cys342Tyr)

GCTCCGTGTCTCTCGGTTGTGGGCTTTGTGGATGGGCTGCAGTCGGAATCTCCCAGTGGCCAGCACCCCCTGAAGCCCCCGGTGCGACGCCTTGTGG TTCCACAGCCCCCTCCACAATCATTCCTGTGTCGTCTAGCCTTTTCTTTTGCTTCCCTTGTTTTCTAGGCCGCCGGTGTTAACACCACGGACAAAGA GATTGAGGTTCTCTATATTCGGAATGTAACTTTTGAGGACGCTGGGGAATATACGTRCTTGGCGGGTAATTCTATTGGGATATCCTTTCACTCTGCA TGGTTGACAGTTCTGCCAGGTATATACTGTTCTTTCTCTCTGGGTTTTTTTCCCTTTTCTTGGTTGACTGCTATAAAATTAACACAGCTTCTGTTAT CAGAAATGGCCCCTTTTATCCTTGCATAAAGATATAAAAAATGTTAAAAATGATCCCTCAGGGATAAGAAAACTGCCTTGGAAATTCACACACAGTG AGATCCCACACTCACA

>6524(ATPLFGALTF)

CTTTTGCTCTTGTTTTCTAGGCCGCCGGTGTTACACCACGGACAAAGAGATTGAGGTTCTCTATATTCGGAATGTAACTTTTGAGGACGCTGGGGAA TATACGTGCTTGGCGGGTAATTCTATTGGGATATCCTTTCACTCTGCATGGTTGACAGTTCTGCCAGGTATATACTGTTCTTTCTCTCTGGGTTTTT TTCCCTTTTCTTGGTTGACTGCTATAAAATTAACACAGCTTCTGTTATCAGAAATGGCCCCTTTTATCCTTGCATAAAGATATAAAAAATGTTAAAA ATGATCCCTCAGGGATAAGAAAACTGCCTTGGAAATTCACACACAGGGAGAATCCCAATAGTAGAATTACCCGCCAAGGAGGTAGTTCTCCGTATTT CCCCCCGGCGTCACTCACTTGTAGGT

>6524(ATPLFGALTR)

CCACAATCCATTCTGTGTTGTCTAGCCTTTTCTTTTGCTTCCCTTGTTTTTTAGGCCGCCGGTGTTAACACCACGGACAAAGAGATTGAGGTTCTCT ATATTCGGAATGTAACTTTTGAGGACGCTGGGGAATATACGTGCTTGGCGGGTAATTCTATTGGGATATCCTTTCACTTTGCATGGTTGACAGTTTT GCCAGGTATATACTGTTCTTTCTCTTTGGGTTTTTTTCCCTTTTCTTGGTTGACTGCTATAAAATTAACACAGCTTCTGTTATCAGAAATGGCCCCT TTTATCCTTGCATAAAGATATAAAAAATGTAAAAATGTCCCTCATGATAGACTC

| ref | CAGCACCCCCTGAAGCCCCCGGTGCGACGCCTTGTGGTTCCACAGCCCCCTCCACAATCA | | | | | |
| --- | --- | --- | --- | --- | --- | --- |
| 6524_ATPLFGALTR_ | --------------------------------------------------- | |  |  |  | CCACAATCC |
| 6524_ATPLFGALTF_ | ------------------------------------------------------------ | |  |  |  |  |
| ref | TTCCTGTGTCGTCTAGCCTTTTCTTTTGCTTCCCTTGTTTTCTAGGCCGCCGGTGTTAAC | | | | | |
| 6524_ATPLFGALTR_ | ATTCTGTGTTGTCTAGCCTTTTCTTTTGCTTCCCTTGTTTTTTAGGCCGCCGGTGTTAAC | | | | | |
| 6524_ATPLFGALTF_ | ---------------------- | |  |  | CTTTTGCT | ---CTTGTTTTCTAGGCCGCCGGTGTTA-C |
|  | ******** | | | | | ******** **************** * |
| ref | ACCACGGACAAAGAGATTGAGGTTCTCTATATTCGGAATGTAACTTTTGAGGACGCTGGG | | | | | |
| 6524_ATPLFGALTR_ | ACCACGGACAAAGAGATTGAGGTTCTCTATATTCGGAATGTAACTTTTGAGGACGCTGGG | | | | | |
| 6524_ATPLFGALTF_ | ACCACGGACAAAGAGATTGAGGTTCTCTATATTCGGAATGTAACTTTTGAGGACGCTGGG | | | | | |
|  | ************************************************************ | | | | | |
| ref | GAATATACGTRCTTGGCGGGTAATTCTATTGGGATATCCTTTCACTCTGCATGGTTGACA | | | | | |
| 6524_ATPLFGALTR_ | GAATATACG | TGC |  |  | TTGGCGGGTAATTCTATTGGGATATCCTTTCACTTTGCATGGTTGACA | |
| 6524_ATPLFGALTF_ | GAATATACGTGCTTGGCGGGTAATTCTATTGGGATATCCTTTCACTCTGCATGGTTGACA | | | | | |
|  | ********** *********************************** ************* | | | | | |
| ref | GTTCTGCCAGGTATATACTGTTCTTTCTCTCTGGGTTTTTTTCCCTTTTCTTGGTTGACT | | | | | |
| 6524_ATPLFGALTR_ | GTTTTGCCAGGTATATACTGTTCTTTCTCTTTGGGTTTTTTTCCCTTTTCTTGGTTGACT | | | | | |
| 6524_ATPLFGALTF_ | GTTCTGCCAGGTATATACTGTTCTTTCTCTCTGGGTTTTTTTCCCTTTTCTTGGTTGACT | | | | | |
|  | *** ************************** ***************************** | | | | | |
| ref | GCTATAAAATTAACACAGCTTCTGTTATCAGAAATGGCCCCTTTTATCCTTGCATAAAGA | | | | | |
| 6524_ATPLFGALTR_ | GCTATAAAATTAACACAGCTTCTGTTATCAGAAATGGCCCCTTTTATCCTTGCATAAAGA | | | | | |
| 6524_ATPLFGALTF_ | GCTATAAAATTAACACAGCTTCTGTTATCAGAAATGGCCCCTTTTATCCTTGCATAAAGA | | | | | |
|  | ************************************************************ | | | | | |
| ref | TATAAAAAATGTTAAAAATGATCCCTCAGGGATAAGAAAACTGCCTTGGAAATTCACACA | | | | | |
| 6524_ATPLFGALTR_ | TATAAAAAATGT-AAAAATG-TCCCTCATG-ATAGACTC--------------------- | | | | | |
| 6524_ATPLFGALTF_ | TATAAAAAATGTTAAAAATGATCCCTCAGGGATAAGAAAACTGCCTTGGAAATTCACACA | | | | | |
|  | ************ ******* ******* * *** | | | | | |

ref CAGTGAGATCCCACACTCACA---------------------------------------

6524_ATPLFGALTR_ ------------------------------------------------------------

6524_ATPLFGALTF_ CAGGGAGAATCCCAATAGTAGAATTACCCGCCAAGGAGGTAGTTCTCCGTATTTCCCCCC

TGC 342


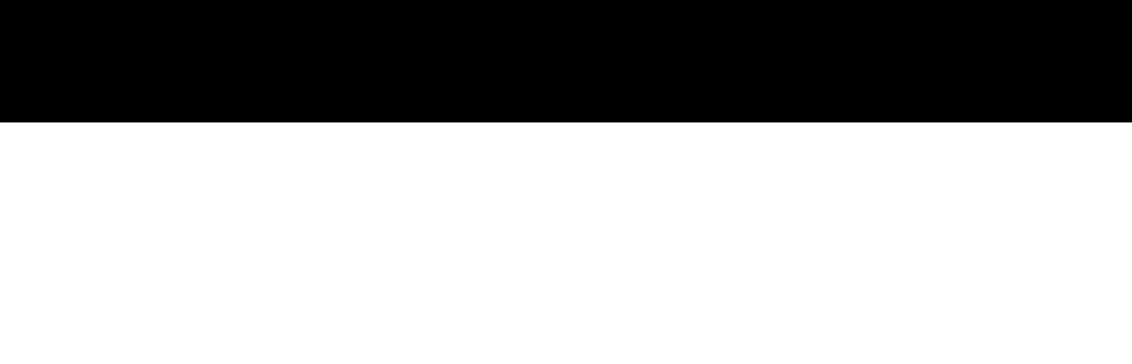

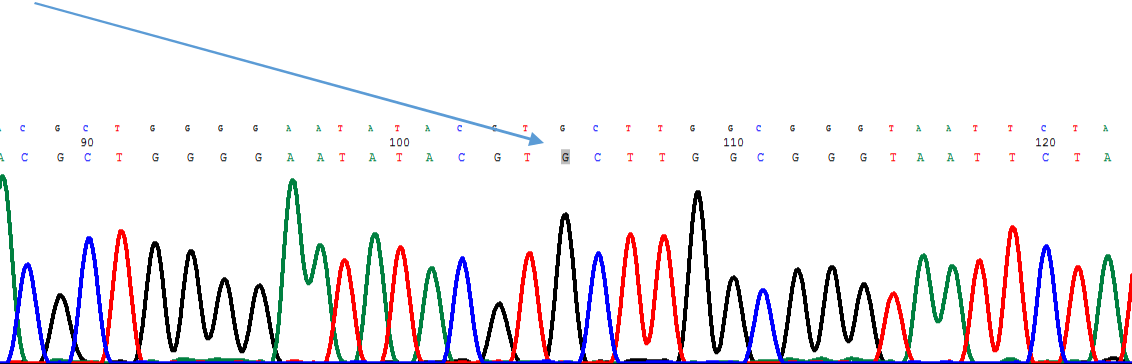


GCG 344

TCT 347


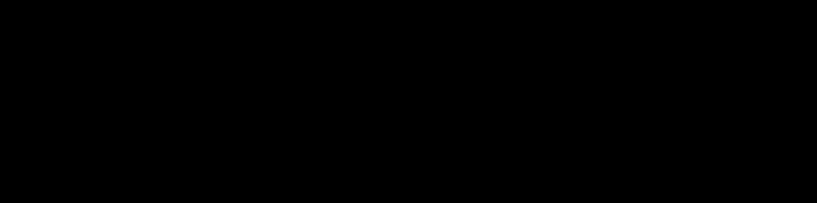

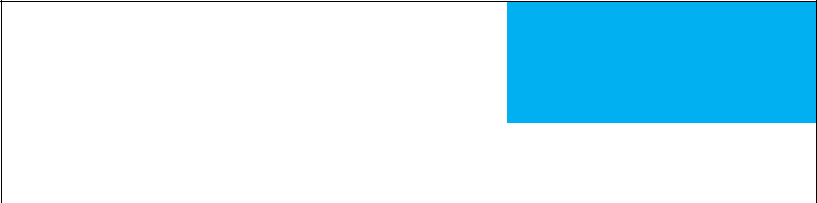


| Sample F | FGFR2 |  |
| --- | --- | --- |
|  |  | C342S (G-C) |
|  |  |  |
|  |  |  |
|  |  | C342Y (G-A) |

C342W (C-G)

At C342 position codon TGC (Amino acid C) observed hence no mutation detected.


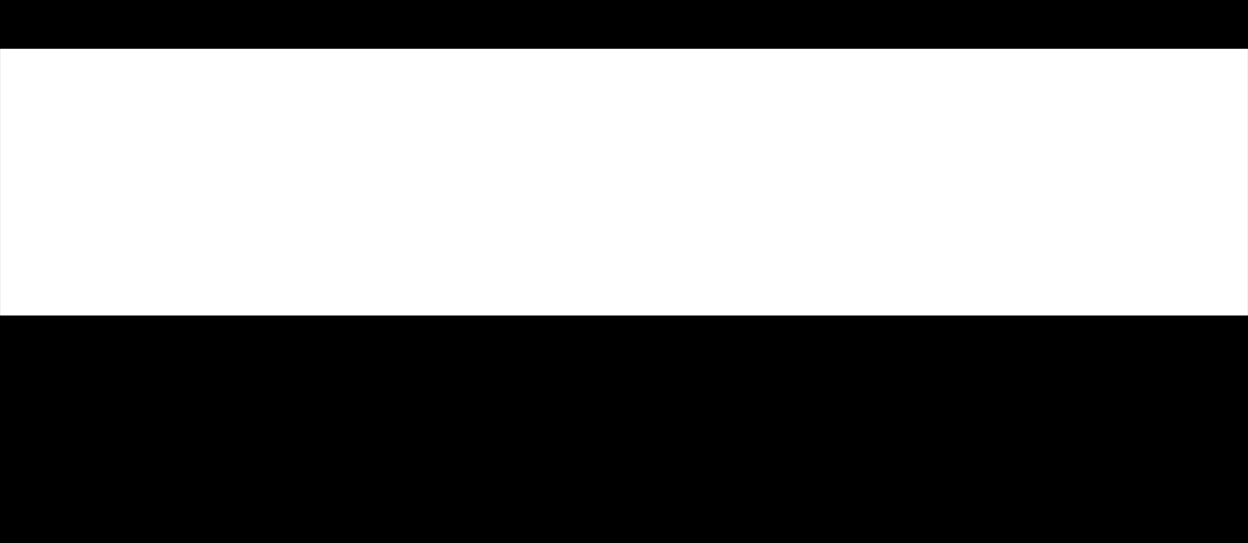

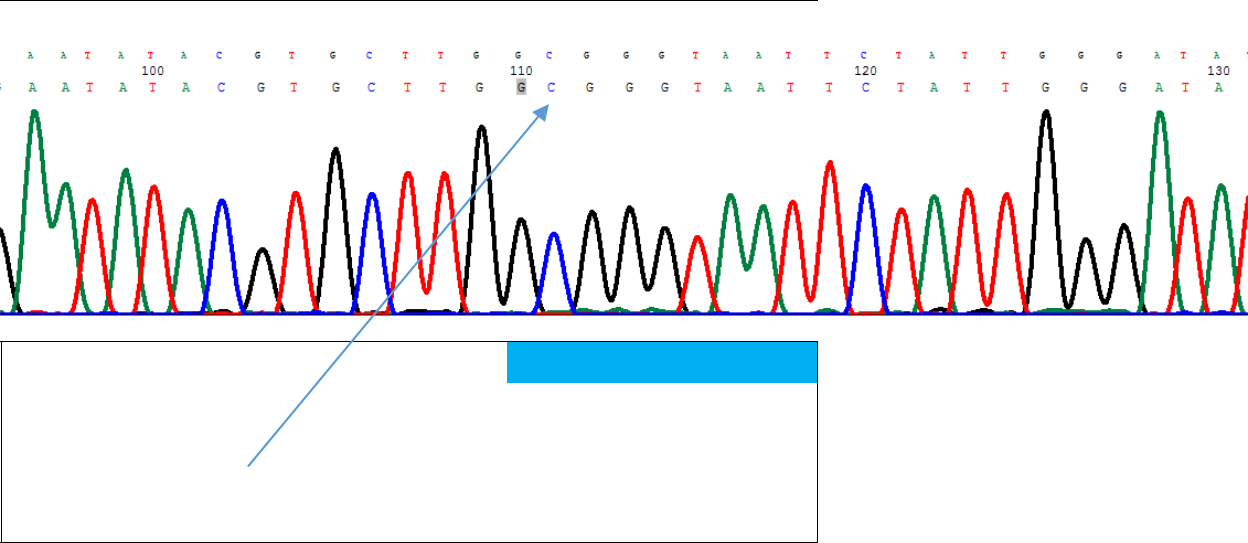


| Sample F | FGFR2 | A344A (G-A) |
| --- | --- | --- |
|  |

At A344 position codon GCG (Amino acid A) observed WITHOUT NONSYNONYMOUS CHANGE OF NUCLEOTIDE hence no mutation detected. GCG^🡪^GCA NOT DETECTED


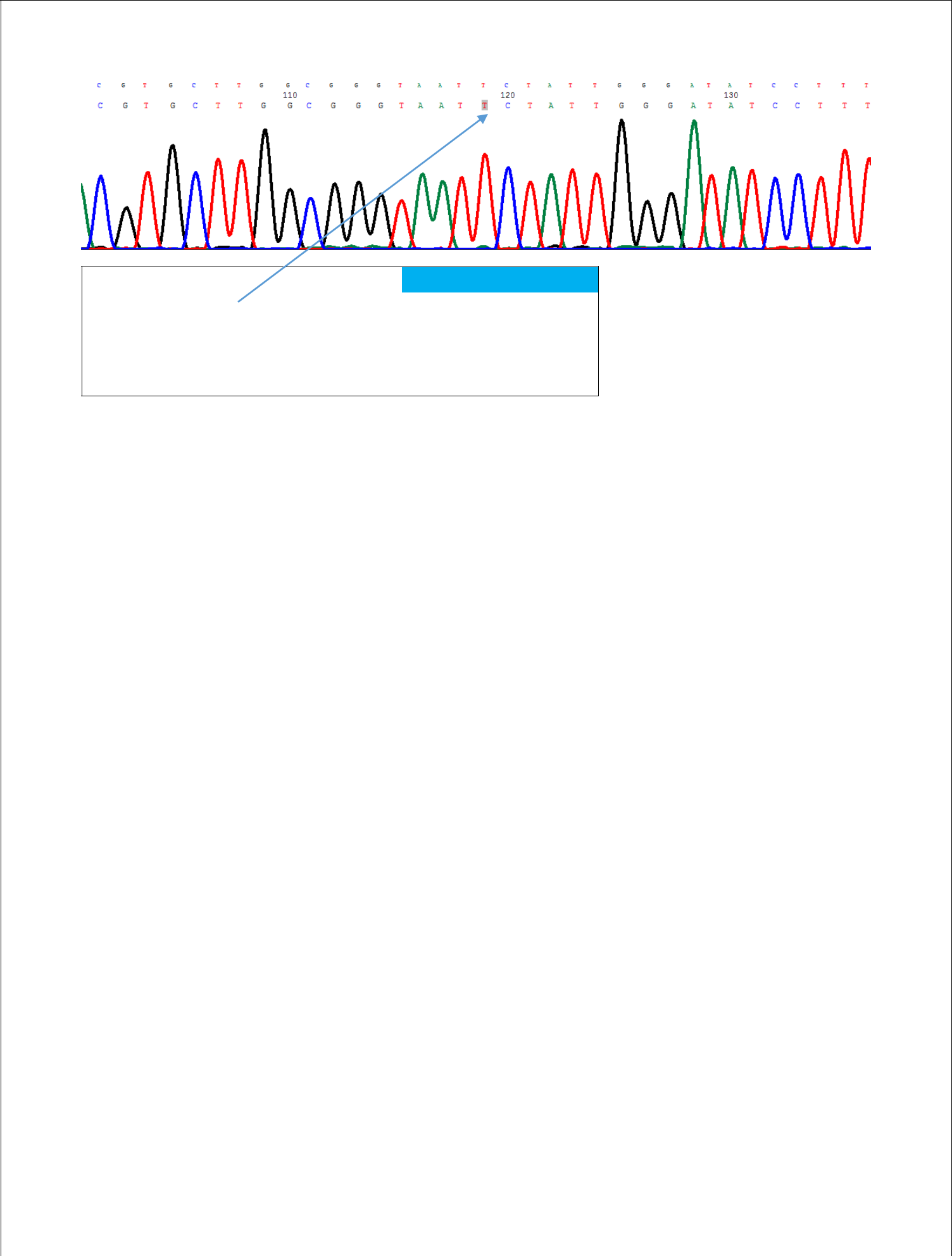


| Sample F | FGFR2 | S347C (C-G) |
| --- | --- | --- |
|  |

At S347 position codon TCT (Amino acid S) observed. Hence no mutation detected.


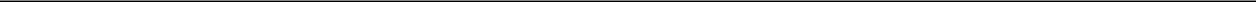

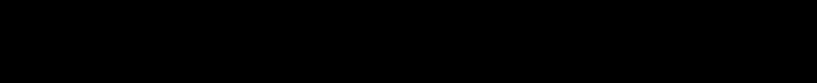

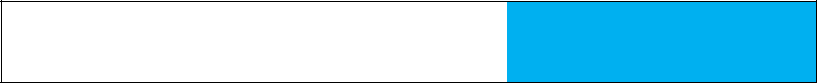


| **Sample D** | **STRA6** | T>C P.Y374C |
| --- | --- | --- |
|  |

>STRA6 T>C P.Y374C

CTCTGTTCTCGTCCACACAGTCCTCACCTCCTATCTCCTTCCATTCCCGGGCAGGTGCAGCTTCCCCAGGCCACCTTCCCTCTCATCCAGCCCACCC GGTGGCCCCTACTCAGGGGGCAAGCTCCACTAGCTGCCCTGAGTTGGTGGTGCCCTATGTCCCTCACACTCACAGACGTTTCCCCAACCAGACAGGA AGGCCCAAGGGCAGGACCCAGCCCCACCTTCTCCCACTGCAGCGCAGCCACGCACAGGTGGGCTCCATGACAGCCCGGGCCGGCAGAGCCCTTCCCT CCCTCCAGGCCCAGGGCCTCCCCGCAGGCCCACAGGACTCCCACTCCTTCCCCACCTCGGCGCTGGGTGAGCCAGAGTCTGTCACTCACCTGTGTGT CACCAGTGAGCGCATCAGGACCAGGAAGGTGAGTAAGCAGGACAAGACCAAGGCTGAGATGTAGCACACTGGTGGGCAGAGAATGAGCAAAGTGAGA GGTCAGGGTCTGGAGAGTCTCCTGGAGGCCTCAGAACCCCTGCCAGGGTGGTGCCTTGTGGGGAGTTCCCCCTGCCCCAAACTGAACTCTGTGTGGA AGCCTCTTGGCCCCACCCCCAGGTCTCAGATGCAGTGTAAGCCAATGACTTCGGCCACCCCATTCTTCC

>6521A(ST1)

CACATATCTTTCCTCCTCAGCCCAGACTCGCGCAGGCCCACAGGACTCCCACTCCTTCCCCACCTCGGCCCTGTGTGAGCCAGAGTCTGTCACTCAC

CTGTGTGTCACCAGTGAGCGCATCAGGACCAGGAAGGTGAGTAAGCAGGACAAGACCAAGGCTGAGATGTACCACACTGGTGGGCAGAGAATGAGCA

AAGTGAGAGGTCAGGGTCTGGAGAGTCTCCTGGAGGCCTCAGAACCCCTGCCAGGGTGGTGCCTTGTGGGGAGTTCCCCCTGCCCCAAACTGAACTC

TGTGTGGAAGCCTCTTGGCCCCACCCCCAGGTCTCAGATGCAGTGTAAGCCAATGATAAA

>6521A(ST2)

GCCCTTCCCTCCCTCCAAGCCCAGGGCCTCCCCGCAGGCCCACAGGACTCCCACTCCTTCCCCACCTCGGCGCTGGGTGAGCCAGAGTCTGTCACTC

ACCTGTGTGTCACCAGTGAGCGCATCAGGACCAGGAAGGTGAGTAAGCAGGACAAGACCAAGGCTGAGATGTAGCACACTGGTGGGCAGAGAATGAG

CAAAGTGAGAGGTCAGGGTCTGGAGAGTCTCCTGGAGGCCTCAGAACCCCTGCCAGGGTGGTGCCTTGTGGGGAGTTCCCCCTGCCCCAAACTGAAC

TCTGTGGGAAGCCTCTGGCACGCCAAAGGGGCCCCT

straref CAAGAGGCTTCCACACAGAGTTCAGTTTGGGGCAGGGGGAACTCCCCACAAGGCACCACC

6521A_ST2_ CA-GAGGCTTCC-CACAGAGTTCAGTTTGGGGCAGGGGGAACTCCCCACAAGGCACCACC

6521A_ST1_ CAAGAGGCTTCCACACAGAGTTCAGTTTGGGGCAGGGGGAACTCCCCACAAGGCACCACC

** ********* ***********************************************

straref CTGGCAGGGGTTCTGAGGCCTCCAGGAGACTCTCCAGACCCTGACCTCTCACTTTGCTCA

6521A_ST2_ CTGGCAGGGGTTCTGAGGCCTCCAGGAGACTCTCCAGACCCTGACCTCTCACTTTGCTCA

6521A_ST1_ CTGGCAGGGGTTCTGAGGCCTCCAGGAGACTCTCCAGACCCTGACCTCTCACTTTGCTCA

************************************************************

| straref | TTCTCTGCCCACCAGTGTGCTACATCTCAGCCTTGGTCTTGTCCTGCTTACTCACCTTCC | | |
| --- | --- | --- | --- |
| 6521A_ST2_ | TTCTCTGCCCACCAGTGTGCTACATCTCAGCCTTGGTCTTGTCCTGCTTACTCACCTTCC | | |
| 6521A_ST1_ | TTCTCTGCCCACCAGTGTGGTACATCTCAGCCTTGGTCTTGTCCTG | C | TTACTCACCTTCC |
|  | ******************* **************************************** | | |
| straref | TGGTCCTGATGCGCTCACTGGTGACACACAGGTGAGTGACAGACTCTGGCTCACCCAGCG | | |
| 6521A_ST2_ | TGGTCCTGATGCGCTCACTGGTGACACACAGGTGAGTGACAGACTCTGGCTCACCCAGCG | | |
| 6521A_ST1_ | TGGTCCTGATGCGCTCACTGGTGACACACAGGTGAGTGACAGACTCTGGCTCACACAGGG | | |
|  | ****************************************************** *** * | | |
| straref | CCGAGGTGGGGAAGGAGTGGGAGTCCTGTGGGCCTGCGGGGAGGCCCTGGGCCTGGAGGG | | |
| 6521A_ST2_ | CCGAGGTGGGGAAGGAGTGGGAGTCCTGTGGGCCTGCGGGGAGGCCCTGGGCTTGGAGGG | | |
| 6521A_ST1_ | CCGAGGTGGGGAAGGAGTGGGAGTCCTGTGGGCCTGCGCGAG---TCTGGGCTGAGGAGG | | |
|  | ************************************** * | ****** * ** | |


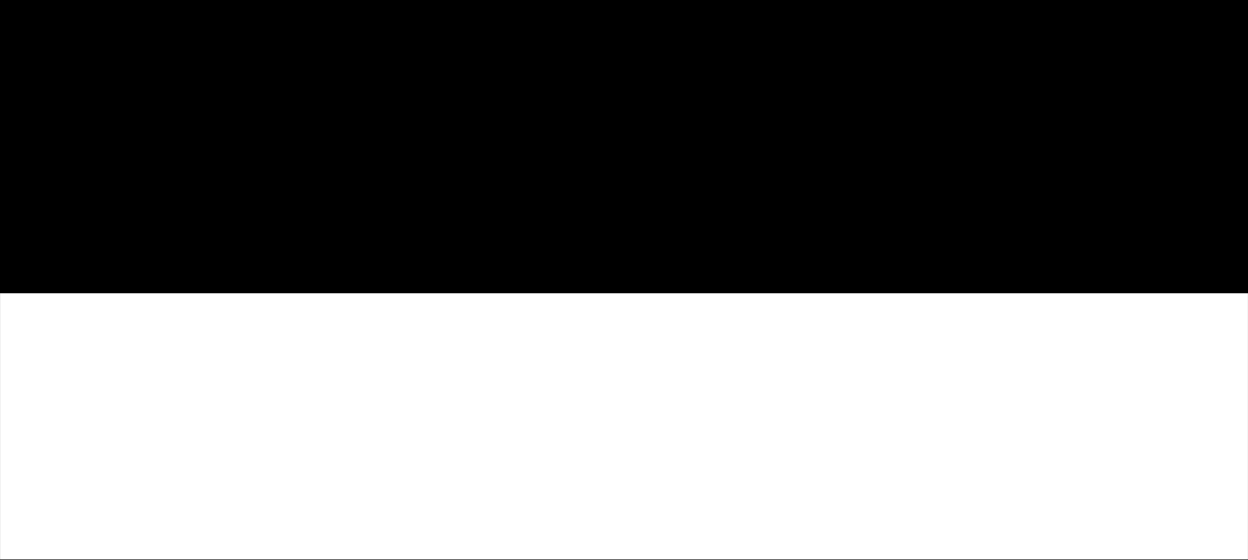

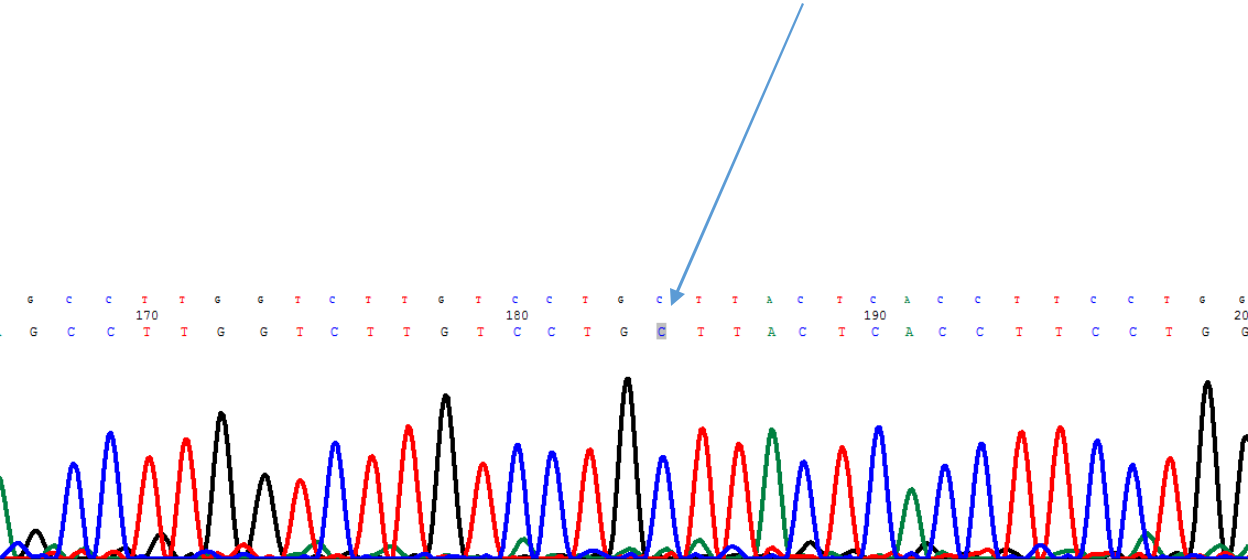


| **Sample D** |  | A>T P.L152M |
| --- | --- | --- |
|  | **STRA6** |  |
|  |  |  |

>STRA6 A>T P.L152M

ATGCATATTAGCCCCATTTTGCAGTGGAGGAACTGAGAGCCAGGGAAGTAGTATCTTAAATTGAGTATTTGCTCAGGCACCATGCTAAGCATTATAT

ATCCACCTTCTCATTTTGCCCTTGCTAATATACTGTGATTATTTTCATTTTACAGATGGGAAAACTGAGGCTCCAAGAGGTAACTTGCCCAAGATCA

CAGAGATGAGACTAGTCAGAGCCTGGATTTGAACCTAGTGTTCTTGCCCACTGAACGTGCTGAAGGGCTTGGAAGCTGGAGACCGAAGAGAGGCCTG

GGTGGGCTCACTGCAGGCCCCATGTCTGCCCTTCCAGCATGGGTCTCTCTGCTTGTTTCACCTTCCAGGGGCCTGGAAGATACTGGGACTGTTCTAT

TATGCTGCCCTCTACTACCCTCTGGCTGCCTGTGCCACGGCTGGCCACACAGCTGCACACCTGCTCGGCAGCACGCTGTCCTGGGCCCACCTTGGGG

TCCAGGTCTGGCAGAGGGCAGAGTGTCCCCAGGTGCCCAAGGTAACCGCTGATCACAATGGCCTANGGCAGAGGGGCGCACAGAGCAACCTACTCAA

ACCACAGTGCCTNCTTTGAGCTCTGATCTATGA

>6521B(ST3)

CACCGGCAACTTGGCAAGTGGTATCTTAAATTGAGTATTTGCTCAGGCACCATGCTAAGCATTATATATCCACCTTCTCATTTTGCCCTTGCTAATA

TACTGTGATTATTTTCATTTTACAGATGGGAAAACTGAGGCTCCAAGAGGTAACTTGCCCAAGATCACAGAGTTGAGACTAGTCAGAGCCTGGATTT

GAACCTAGTGTTCTTGCCCACTGAACGTGCTGAAGGGCTTGGAAGCTGGAGACCGAAGAGAGGCCTGGGTGGGCTCACTGCAGGCCCCATGTCTGCC

CCTCCAGCATGGGTCTCTCTGCTTGTTTCACCTTCCAGGGGCCTGGAAGATACTGGGACTGTTCTATTATGCTGCCCTCTACTACCCTCCAAA

>6521B(ST4)

TTATTAGCCCCCATTTTGCAATGGAGGAACTGAGAGCCAGGGAAGTAGTATCTTAAATTGAGTATTTGCTCAGGCACCATGCTAAGCATTATATATC CACCTTCTCATTTTGCCCTTGCTAATATACTGTGATTATTTTCATTTTACAGATGGGAAAACTGAGGCTCCAAGAGGTAACTTGCCCAAGATCACAG AGTTGAGACTAGTCAGAGCCTGGATTTGAACCTAGTGTTCTTGCCCACTGAACGTGCTGAAGGGCTTGGAAGCTGGAGACCGAAGAGAGGCCTGGGT GGGCTCACTGCAGGCCCCATGTCTGCCCCTCCAGCATGGGTCTCTCTGCTGTTCACCTCCAGGGCCTGAAGATAACCTACAGGCG

| STRA6 | ATGCATATTAGCCCCATTTTGCAGTGGAGGAACTGAGAGCCAGGGAAGTAGTATCTTAAA |
| --- | --- |
| 6521B_ST3_ | ------------------------------CACCGGCAACTTGGCAAGTGGTATCTTAAA |
| 6521B_ST4_ | ---TTATTAGCCCCCATTTTGCAATGGAGGAACTGAGAGCCAGGGAAGTAGTATCTTAAA |
|  | ** * * * ** **** ********** |
| STRA6 | TTGAGTATTTGCTCAGGCACCATGCTAAGCATTATATATCCACCTTCTCATTTTGCCCTT |
| 6521B_ST3_ | TTGAGTATTTGCTCAGGCACCATGCTAAGCATTATATATCCACCTTCTCATTTTGCCCTT |
| 6521B_ST4_ | TTGAGTATTTGCTCAGGCACCATGCTAAGCATTATATATCCACCTTCTCATTTTGCCCTT |
|  | ************************************************************ |
| STRA6 | GCTAATATACTGTGATTATTTTCATTTTACAGATGGGAAAACTGAGGCTCCAAGAGGTAA |
| 6521B_ST3_ | GCTAATATACTGTGATTATTTTCATTTTACAGATGGGAAAACTGAGGCTCCAAGAGGTAA |
| 6521B_ST4_ | GCTAATATACTGTGATTATTTTCATTTTACAGATGGGAAAACTGAGGCTCCAAGAGGTAA |
|  | ************************************************************ |
| STRA6 | CTTGCCCAAGATCACAGAGATGAGACTAGTCAGAGCCTGGATTTGAACCTAGTGTTCTTG |

| 6521B_ST3_ | CTTGCCCAAGATCACAGAGTTGAGACTAGTCAGAGCCTGGATTTGAACCTAGTGTTCTTG | | |
| --- | --- | --- | --- |
| 6521B_ST4_ | CTTGCCCAAGATCACAGAG | TTG | AGACTAGTCAGAGCCTGGATTTGAACCTAGTGTTCTTG |
|  | ******************* **************************************** | | |
| STRA6 | CCCACTGAACGTGCTGAAGGGCTTGGAAGCTGGAGACCGAAGAGAGGCCTGGGTGGGCTC | | |
| 6521B_ST3_ | CCCACTGAACGTGCTGAAGGGCTTGGAAGCTGGAGACCGAAGAGAGGCCTGGGTGGGCTC | | |
| 6521B_ST4_ | CCCACTGAACGTGCTGAAGGGCTTGGAAGCTGGAGACCGAAGAGAGGCCTGGGTGGGCTC | | |
|  | ************************************************************ | | |
| STRA6 | ACTGCAGGCCCCATGTCTGCCCTTCCAGCATGGGTCTCTCTGCTTGTTTCACCTTCCAGG | | |
| 6521B_ST3_ | ACTGCAGGCCCCATGTCTGCCCCTCCAGCATGGGTCTCTCTGCTTGTTTCACCTTCCAGG | | |
| 6521B_ST4_ | ACTGCAGGCCCCATGTCTGCCCCTCCAGCATGGGTCTCTCTGCTG--TTCACCT-CCAGG | | |
|  | ********************** ********************* ******* ***** | | |


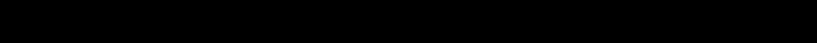

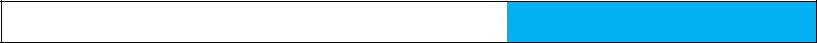

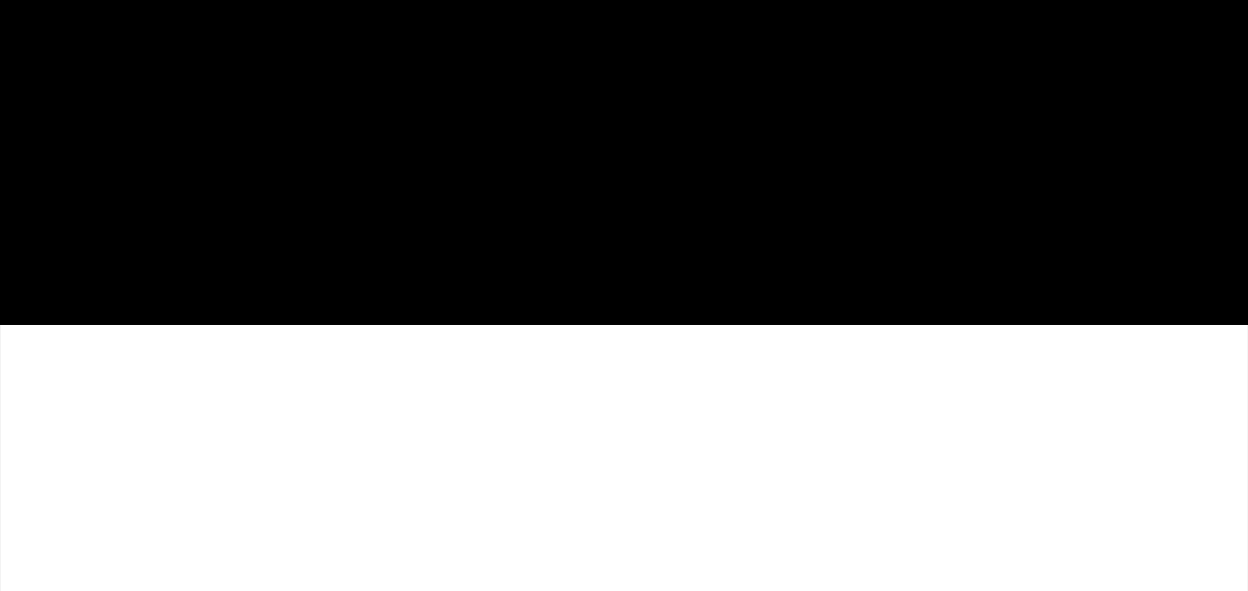

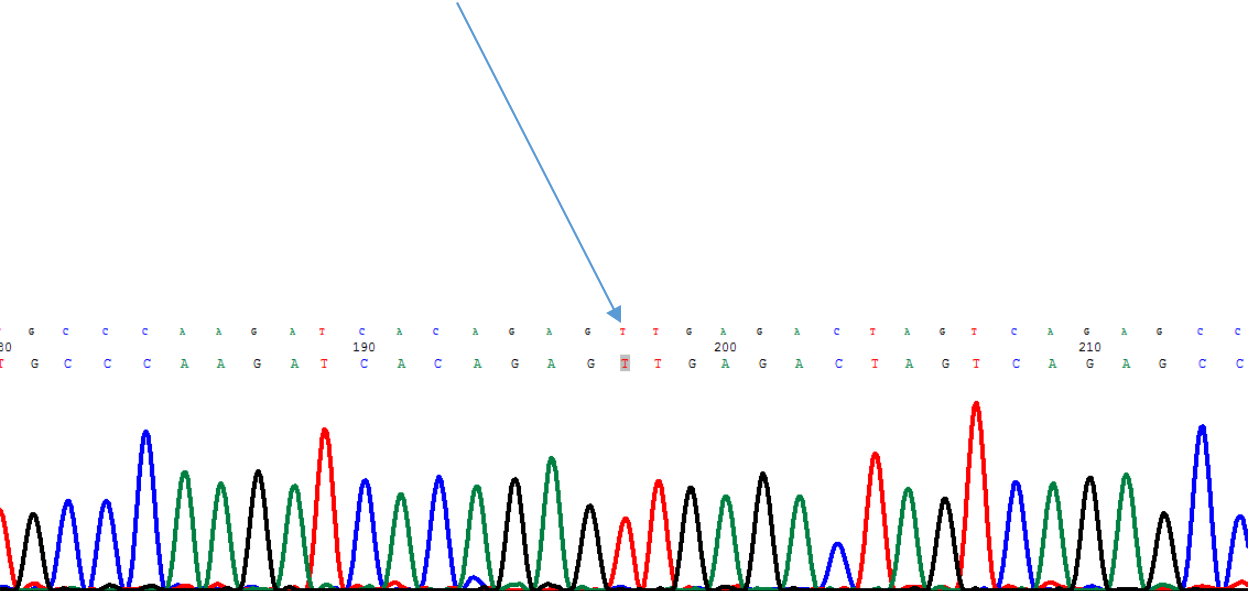


| **Sample D** | **CRYBA4** | T>A P.F25L |
| --- | --- | --- |
|  |

- **CRYBA4**

CCAGATCTAGTGATTGTCTTGAGAAATTGGAAGGTCTGGCCTGCATTCCCACTTGGCAACCATTGGTGGCACTGGGAAGAGGCCACCCATTTACCTG GCACCTGTGCTGTCTAGTGTCTCACATTTATCACTGCTCTTGCCTTCCTGGCTCCTGGAGGAATCTGAGTTTGCAATCCCTGCTTTACCTGCCAGAT CCTGGGGCGAGCCCCCAACCTCTCACCCTTCACGGGACTCTGATGCGGATCTCCACCTTTTTTTTTTCCTGGCACAGATGGTGGTGTGGGATGAGGA CGGCTTCCAGGGCCGGCGGCACGAGTTCACGGCCGAGTGCCCCAGCGTGCTGGAGCTTGGCTTCGAGACTGTGCGATCTTTGAAAGTGCTGAGTGGA GCGTGAGTCTAGGGGGACACTGAGTTGGGGTAGAGGGTGGACAGGAAGGGACCTAGAGACGGGTGCTAGGACTTTTAGATATTCTAGGTCCCCTCTC CCTAGGCTCTTACTGTTGTGCCCTCCTGAAGTACTGAGGAGTGTGCAGGACTGCCATGTAAGATTATGCAGGTTGCGCACTGCCCAACAGTAGGAGG GTGCCATTTACACAGACCC

>6521C(CR1)

CCGTCCTACTGCAGATCCTGGGGCGAGCCCCCAACCTCTCACCCTTCACGGGACTCTGATGCGGATCTCCACCTTTTTTTTTTCCTGGCACAAATGG

TGGTGTGGGATGAGGACGGCTTCCAGGGCCGGCGGCACAAGTTCACGGCCGAGTGCCCCACCGTGCTGGAGCTTGGCTTCAAAACTGTGCAATCTTT

GAAAGTGCTGAGTGGAGCGTGAGTCTAGGGGGACACTGAGTTGGGGTAAAGGGTGGACAGGAAGGGACCTAAAGACGGGTGCTAGGACTTTAAA

>6521C(CR2)

TTTTTCTCCTGGAGGAATTTGAGTTTGCAATCCCTGCTTTACCTGCCAGATCCTGGGGCGAGCCCCCAACCTTTCACCCTTCACGGGACTTTGATGC GGATTTCCACCTTTTTTTTTTCCTGGCACAGATGGTGGTGTGGGATGAGGACGGCTTCCAGGGCCGGCGGCACGAGTTCACGGCCGAGTGCCCCAGC GTGCTGGAGCTTGGCTTCGAGACTGTGCGATCTTTGAAAGTGCTGAGTGGAGCGTGAGTTCTAGGGGGACACTGAGTTTGGTTAGAAGGGGGAAAAA CT

CRYBA4 TTTACCTGCCAGATCCTGGGGCGAGCCCCCAACCTCTCACCCTTCACGGGACTCTGATGC

6521C_CR1_ CCTACTG--CAGATCCTGGGGCGAGCCCCCAACCTCTCACCCTTCACGGGACTCTGATGC

6521C_CR2_ TTTACCTGCCAGATCCTGGGGCGAGCCCCCAACCTTTCACCCTTCACGGGACTTTGATGC

- ************************** ***************** ******

CRYBA4 GGATCTCCACCTTTTTTTTTTCCTGGCACAGATGGTGGTGTGGGATGAGGACGGCTTCCA

6521C_CR1_ GGATCTCCACCTTTTTTTTTTCCTGGCACAAATGGTGGTGTGGGATGAGGACGGCTTCCA

6521C_CR2_ GGATTTCCACCTTTTTTTTTTCCTGGCACAGATGGTGGTGTGGGATGAGGACGGCTTCCA

**** ************************* *****************************

CRYBA4 GGGCCGGCGGCACGAGTTCACGGCCGAGTGCCCCAGCGTGCTGGAGCTTGGCTTCGAGAC

6521C_CR1_ GGGCCGGCGGCACAAGTTCACGGCCGAGTGCCCCACCGTGCTGGAGCTTGGCTTCAAAAC

6521C_CR2_ GGGCCGGCGGCACGAGTTCACGGCCGAGTGCCCCAGCGTGCTGGAGCTTGGCTTCGAGAC


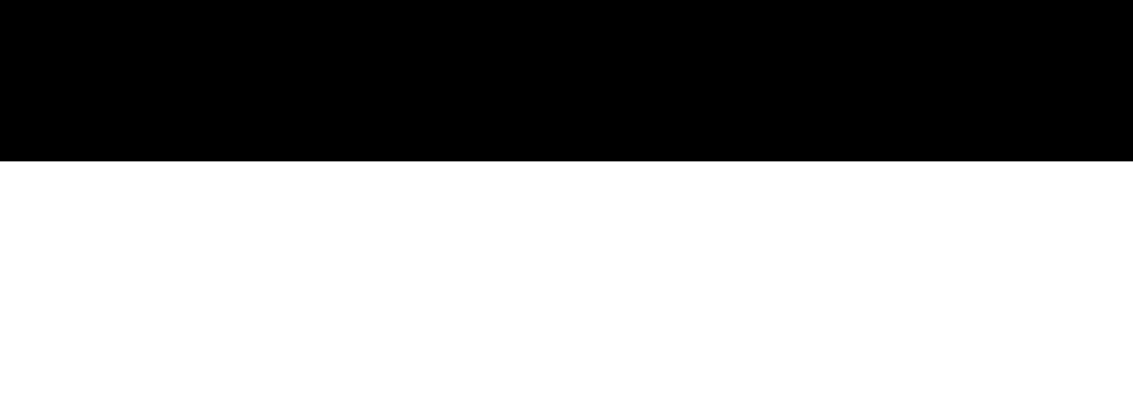

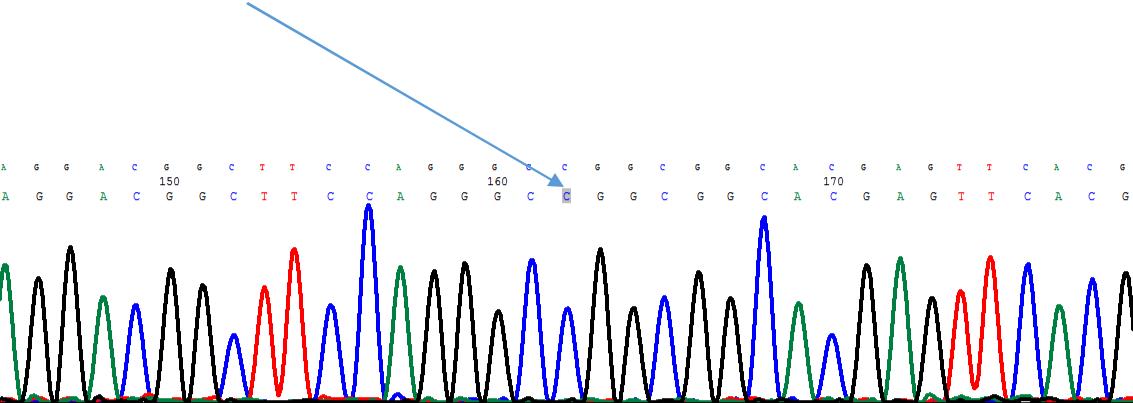


************* ********************* ******************* * **

CRYBA4 TGTGCGATCTTTGAAAGTGCTGAGTGGAGCGTGAGT-CTAGGGGGACACTGAGTTGGGGT

6521C_CR1_ TGTGCAATCTTTGAAAGTGCTGAGTGGAGCGTGAGT-CTAGGGGGACACTGAGTTGGGGT

6521C_CR2_ TGTGCGATCTTTGAAAGTGCTGAGTGGAGCGTGAGTTCTAGGGGGACACTGAGTTTGGTT

***** ****************************** ****************** ** *


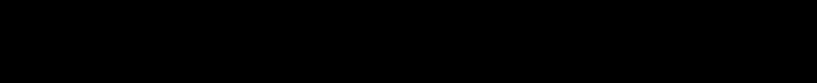

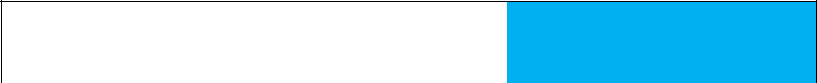


| **Sample D** | **OTX2** | p. Gln104 X |
| --- | --- | --- |
|  |

p. Gln106 His

>OTX2

AGACAGTGTCAAGTGTTTTTAGACAGAGCCTCCCCAACTTTCTTACAAGTCCAGGAGTTTATATGAGAGTACCACATAATAGGTCTTCAGTGGCAGG

GGAAATTGTGTGTTTAGCTGATCTGCCCATGTAGGATAGATTTATAATACGGGAGCCATTCTTGTCCTTAAGGAACTATCAAAACCGAGTTAAAGAA

TTTTCTTTCCCTTCCAAGGTATGGTTTAAGAATCGAAGAGCTAAGTGCCGCCAACAACAGCAACAACAGCAGAATGGAGGTCAAAACAAAGTGAGAC

CTGCCAAAAAGAAGACATCTCCAGCTCGGGAAGTGAGTTCAGAGAGTGGAACAAGTGGCCAATTCACTCCCCCCTCTAGCACCTCAGTCCCGACCAT

TGCCAGCAGCAGTGCTCCTGTGTCTATCTGGAGCCCAGCTTCCATCTCCCCACTGTCAGATCCCTTGTCCACCTCCTCTTCCTGCATGCAGAGGTCC

TATCCCATGACCTATA

>6521D(OT2)

TTTTTGCCTCCCCAACTTTCTTACAAGTCCAGGAGTTTATATGAGAGTACCACATAATAGGTCTTCAGTGGCAGGGGAAATTGTGTGTTTAGCTGAT

CTGCCCATGTAGGATAGATTTATAATACGGGAGCCATTTTTGTCCTTAAGGAACTATCAAAACCGAGTTAAAGAATTTTCTTTCCCTTCCAAGGTAT

GGTTTAAGAATCGAAGAGCTAAGTGCCGCCAACAACAGCAACAACAGCAGAATGGAGGTCAAAACAAAGTGAGACCTGCCAAAAAGAAGACATCTCC

AGCTCGGGAAGTGAGTTCAGAGAGTGGAACAAGTGGCCAATTCACTCCCCCCTCTAGCACCTCATCCCGACCATGCCAGGAGGAATGGGTCG

| OTX2 | AGACAGTGTCAAGTGTTTTTAGACAGAGCCTCCCCAACTTTCTTACAAGTCCAGGAGTTT | |
| --- | --- | --- |
| 6521D_OT2_ | ---------------TTTTT------- | GCCTCCCCAACTTTCTTACAAGTCCAGGAGTTT |
|  | ***** | ********************************* |
| OTX2 | ATATGAGAGTACCACATAATAGGTCTTCAGTGGCAGGGGAAATTGTGTGTTTAGCTGATC | |
| 6521D_OT2_ | ATATGAGAGTACCACATAATAGGTCTTCAGTGGCAGGGGAAATTGTGTGTTTAGCTGATC | |
|  | ************************************************************ | |
| OTX2 | TGCCCATGTAGGATAGATTTATAATACGGGAGCCATTCTTGTCCTTAAGGAACTATCAAA | |
| 6521D_OT2_ | TGCCCATGTAGGATAGATTTATAATACGGGAGCCATTTTTGTCCTTAAGGAACTATCAAA | |
|  | ************************************* ********************** | |
| OTX2 | ACCGAGTTAAAGAATTTTCTTTCCCTTCCAAGGTATGGTTTAAGAATCGAAGAGCTAAGT | |
| 6521D_OT2_ | ACCGAGTTAAAGAATTTTCTTTCCCTTCCAAGGTATGGTTTAAGAATCGAAGAGCTAAGT | |
|  | ************************************************************ | |

OTX2

6521D_OT2_

GCCGCCAACAACAGCAACAACAGCAGAATGGAGGTCAAAACAAAGTGAGACCTGCCAAAA GCCGCCAACAACAGCAACAACAGCAGAATGGAGGTCAAAACAAAGTGAGACCTGCCAAAA ************************************************************


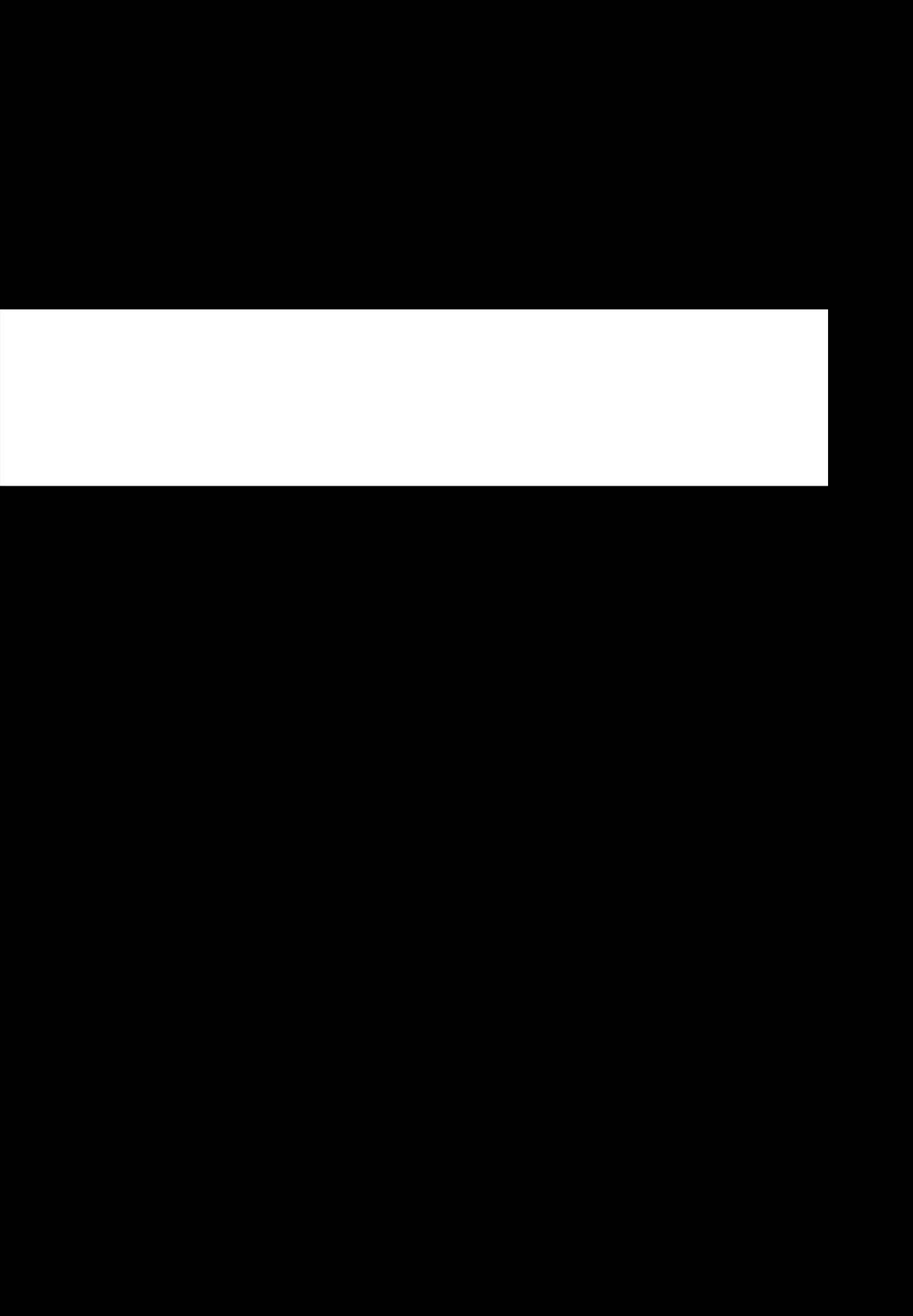

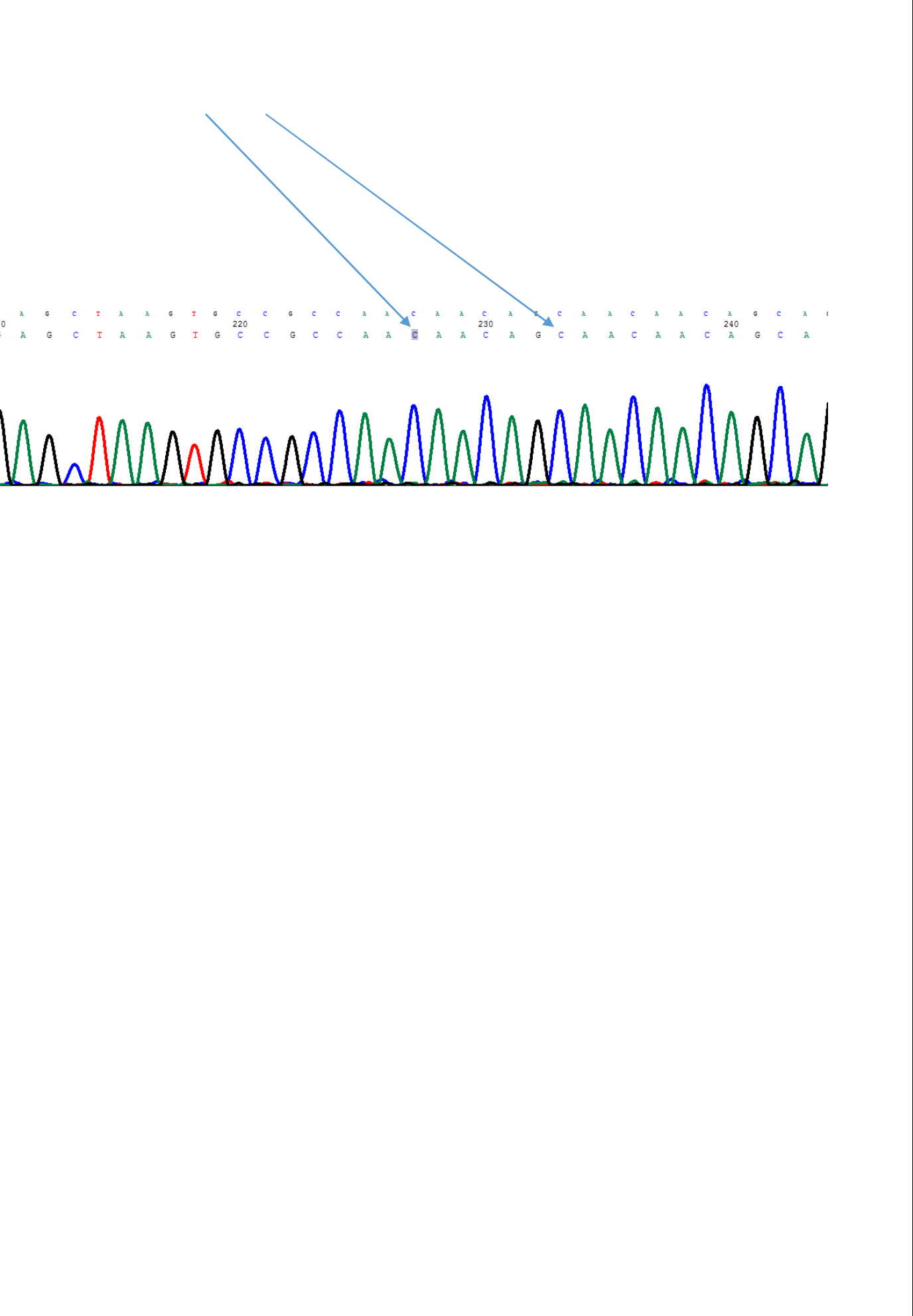


OTX2

6521D_OT2_

AGAAGACATCTCCAGCTCGGGAAGTGAGTTCAGAGAGTGGAACAAGTGGCCAATTCACTC AGAAGACATCTCCAGCTCGGGAAGTGAGTTCAGAGAGTGGAACAAGTGGCCAATTCACTC ************************************************************

OTX2

6521D_OT2_

CCCCCTCTAGCACCTCAGTCCCGACCATTGCCAGCAGCAGTGCTCCTGTGTCTATCTGGA CCCCCTCTAGCACCTCA-TCCCGACCAT-GCCAGGAGGAATGGGTCG------------- ***************** ********** ***** ** * ** *


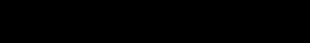

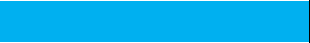


| **Sample D** | **OTX2** | p. Thr186 Fs<frame shift |
| --- | --- | --- |
|  |

>p. Thr186 Fs<frame shift Actual annotation ^🡪^(p.Ser186Asn)

TTCCCTTCCAAGGTATGGTTTAAGAATCGAAGAGCTAAGTGCCGCCAACAACAGCAACAACAGCAGAATGGAGGTCAAAACAAAGTGAGACCTGCCA AAAAGAAGACATCTCCAGCTCGGGAAGTGAGTTCAGAGAGTGGAACAAGTGGCCAATTCACTCCCCCCTCTAGCACCTCAGTCCCGACCATTGCCAG CAGCAGTGCTCCTGTGTCTATCTGGAGCCCAGCTTCCATCTCCCCACTGTCAGATCCCTTGTCCACCTCCTCTTCCTGCATGCAGAGGTCCTATCCC ATGACCTATACTCAGGCTTCAGGTTATAGTCAAGGATATGCTGGCTCAACTTCCTACTTTGGGGGCATGGACTGTGGATCATATTTGACCCCTATGC ATCACCAGCTTCCCGGACCAGGGGCCACACTCAGTCCCATGGGTACCAATGCAGTCACCAGCCATCTCAATCAGTCCCCAGCTTCTCTTTCCACCCA GGGATATGGAGCTTCAAGCTTGGGTTTTAACTCAACCACTGATTGCTTGGATTATAAGGACCAAACTGCCTCCTGGAAGCTTAACTTCAATGCTGAC TGCTTGGATTATAAAGATC

>6521E(OT3)

ACGTGGGGGAAATGGAGTTAGAGAGTGGAACAAGTGGCCAATTCACTCCCCCCTCTAGCACCTCAGTCCCGACCATTGCCAGCAGCAGTGCTCCTGT

GTCTATCTGGAGCCCAGCTTCCATCTCCCCACTGTCAGATCCCTTGTCCACCTCCTCTTCCTGCATGCAGAGGTCCTATCCCATGACCTATACTCAG

GCTTCAGGTTATAGTCAAGGATATGCTGGCTCAACTTCCTACTTTGGGGGCATGGACTGTGGATCATATTTGACCCCTATGCATCACCAGCTTCCCG

GACCAGGGGCCACACTCAGTCCCATGGGTACCAATGCAGTCACCAGCCATCTCAATCAGTCCCCAGCTAAAAA

>6521E(OT4)

TTTTTGAGACCTGCCAAAAAGAAGACATCTCCAGCTCGGGAAGTGAGTTCAGAGAGTGGAACAAGTGGCCAATTCACTCCCCCCTCTAGCACCTCAG TCCCGACCATTGCCAGCAGCAGTGCTCCTGTGTCTATCTGGAGCCCAGCTTCCATCTCCCCACTGTCAGATCCCTTGTCCACCTCCTCTTCCTGCAT GCAGAGGTCCTATCCCATGACCTATACTCAGGCTTCAGGTTATAGTCAAGGATATGCTGGCTCAACTTCCTACTTTGGGGGCATGGACTGTGGATCA TATTTGACCCCTATGCATCACCAGCTTCCCGGACCAGGGGCCACACTCAGTCCCATGGACCAATGAATGAACGG

p.Thr186Fs GAAGTGAGTTCAGAGAGTGGAACAAGTGGCCAATTCACTCCCCCCTCTAGCACCTCAGTC

6521E_OT4_ GAAGTGAGTTCAGAGAGTGGAACAAGTGGCCAATTCACTCCCCCCTCTAGCACCTCAGTC

6521E_OT3_ GAAATGGAGTTAGAGAGTGGAACAAGTGGCCAATTCACTCCCCCCTCTAGCACCTCAGTC

*** ** * *************************************************

p.Thr186Fs CCGACCATTGCCAGCAGCAGTGCTCCTGTGTCTATCTGGAGCCCAGCTTCCATCTCCCCA

6521E_OT4_ CCGACCATTGCCAGCAGCAGTGCTCCTGTGTCTATCTGGAGCCCAGCTTCCATCTCCCCA

6521E_OT3_ CCGACCATTGCCAGCAGCAGTGCTCCTGTGTCTATCTGGAGCCCAGCTTCCATCTCCCCA

************************************************************

p.Thr186Fs CTGTCAGATCCCTTGTCCACCTCCTCTTCCTGCATGCAGAGGTCCTATCCCATGACCTAT

6521E_OT4_ CTGTCAGATCCCTTGTCCACCTCCTCTTCCTGCATGCAGAGGTCCTATCCCATGACCTAT

6521E_OT3_ CTGTCAGATCCCTTGTCCACCTCCTCTTCCTGCATGCAGAGGTCCTATCCCATGACCTAT

************************************************************

p.Thr186Fs ACTCAGGCTTCAGGTTATAGTCAAGGATATGCTGGCTCAACTTCCTACTTTGGGGGCATG

6521E_OT4_ ACTCAGGCTTCAGGTTATAGTCAAGGATATGCTGGCTCAACTTCCTACTTTGGGGGCATG

6521E_OT3_ ACTCAGGCTTCAGGTTATAGTCAAGGATATGCTGGCTCAACTTCCTACTTTGGGGGCATG


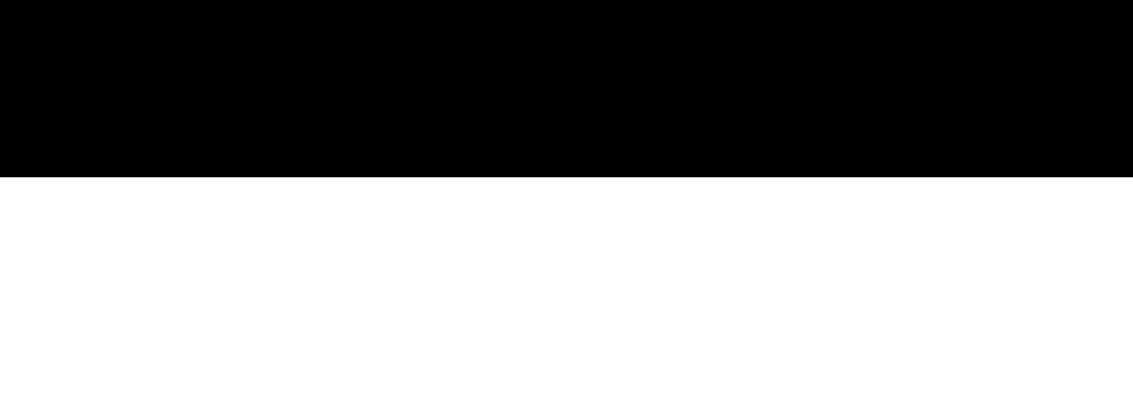

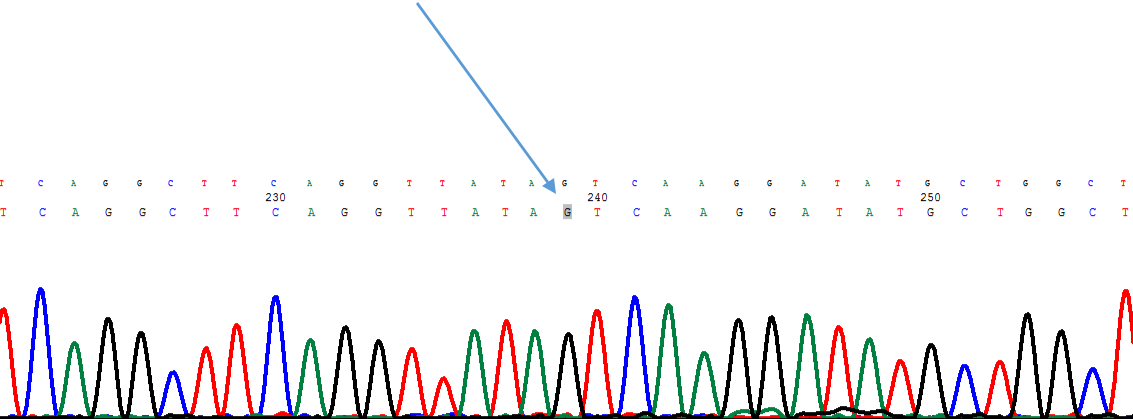


************************************************************

p.Thr186Fs GACTGTGGATCATATTTGACCCCTATGCATCACCAGCTTCCCGGACCAGGGGCCACACTC

6521E_OT4_ GACTGTGGATCATATTTGACCCCTATGCATCACCAGCTTCCCGGACCAGGGGCCACACTC

6521E_OT3_ GACTGTGGATCATATTTGACCCCTATGCATCACCAGCTTCCCGGACCAGGGGCCACACTC

************************************************************


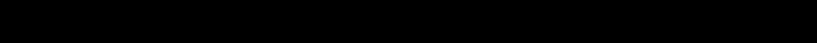

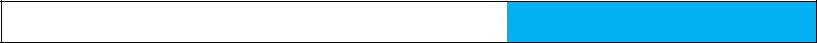


| **Sample C** | **ABCB6** | p. Ala 57 Thr G^🡪^A |
| --- | --- | --- |
|  |

>ABCB6

TCCGGTCCCGCAGTGCCCGCAGCCTCGGCCGGCGTCCACGCATTGCCATGGTGACTGTGGGCAACTACTGCGAGGCCGAAGGGCCCGTGGGTCCGGC

CTGGATGCAGGATGGCCTGAGTCCCTGCTTCTTCTTCACGCTCGTGCCCTCGACGCGGATGGCTCTGGGGACTCTGGCCTTGGTGCTGGCTCTTCCC

TGCAGACGCCGGGAGCGGCCCGCTGGTGCTGATTCGCTGTCTTGGGGGGCCGGCCCTCGCATCTCTCCCTACGTGCTGCAGCTGCTTCTGGCCACAC

TTCAGGCGGCGCTGCCCCTGGCCGGCCTGGCTGGCCGGGTGGGCACTGCCCGGGGGGCCCCACTGCCAAGCTATCTACTTCTGGCCTCCGTGCTGGA

GAGTCTGGCCGGCGCCTGTGGCCTGTGGCTGCTTGTCGTGGAGCGGAGCCAGGCACGGCAGCGTCTGGCAATGGGCATCTGGATCAAGTTCAGGCAC

AGCCCTGGTCTCCTGCTCCTCTGGACTGTGGCGTTTGCAGCTGAGAACTTGGCCCTGGTGTCTTGGAACAGCCCACAGTGGTGGTGGGCAAGGGCAG

ACTTGGGCCAGCAGGTGAGGGACTCTGTGGGAAGGGGGA

>6524(AB1)

CATGTCCTGTAAAGAAATTATGAACTAAGGATCTGGCCTTGGTGCTGGCTCTTCCCTGCAGACGCCGGGAGCGGCCCGCTGGTGCTGATTCGCTGTC

TTGGGGGGCCGGCCCTCGCATCTCTCCCTACGTGCTGCAGCTGCTTCTGGCCACACTTCAGGCGGCGCTGCCCCTGGCCGGCCTGGCTGGCCGGGTG

GGCACTGCCCGGGGGGCCCCACTGCCAAGCTATCTACTTCTGGCCTCCGTGCTGGAGAGTCTGGCCGGCGCCTGTGGCCTGTGGCTGCTTGTCGTGG

AGCGGAGCCAGGCACGGCAGCGTCTGGCAATGGGCATCTGGATCAAGTTA

>6524(AB2)

CCTGAGTCCCTGCTTCTTCTTCACGCTCGTGCCCTCGACGCGGATGGCTCTGGGGACTCTGGCCTTGGTGCTGGCTCTTCCCTGCAGACGCCGGGAG CGGCCCGCTGGTGCTGATTCGCTGTCTTGGGGGGCCGGCCCTCGCATCTCTCCCTACGTGCTGCAGCTGCTTCTGGCCACACTTCAGGCGGCGCTGC CCCTGGCCGGCCTGGCTGGCCGGGTGGGCACTGCCCGGGGGGCCCCACTGCCAAGCTATCTACTTCTGGCCTCCGTGCTGGAGAGTCTGGCCGGCGC CCTGTGGGTCC

ABCB6 GGTGCTGGCTCTTCCCTGCAGACGCCGGGAGCGGCCCGCTGGTGCTGATTCGCTGTCTTG

6524_AB2_ GGTGCTGGCTCTTCCCTGCAGACGCCGGGAGCGGCCCGCTGGTGCTGATTCGCTGTCTTG

6524_AB1_ GGTGCTGGCTCTTCCCTGCAGACGCCGGGAGCGGCCCGCTGGTGCTGATTCGCTGTCTTG


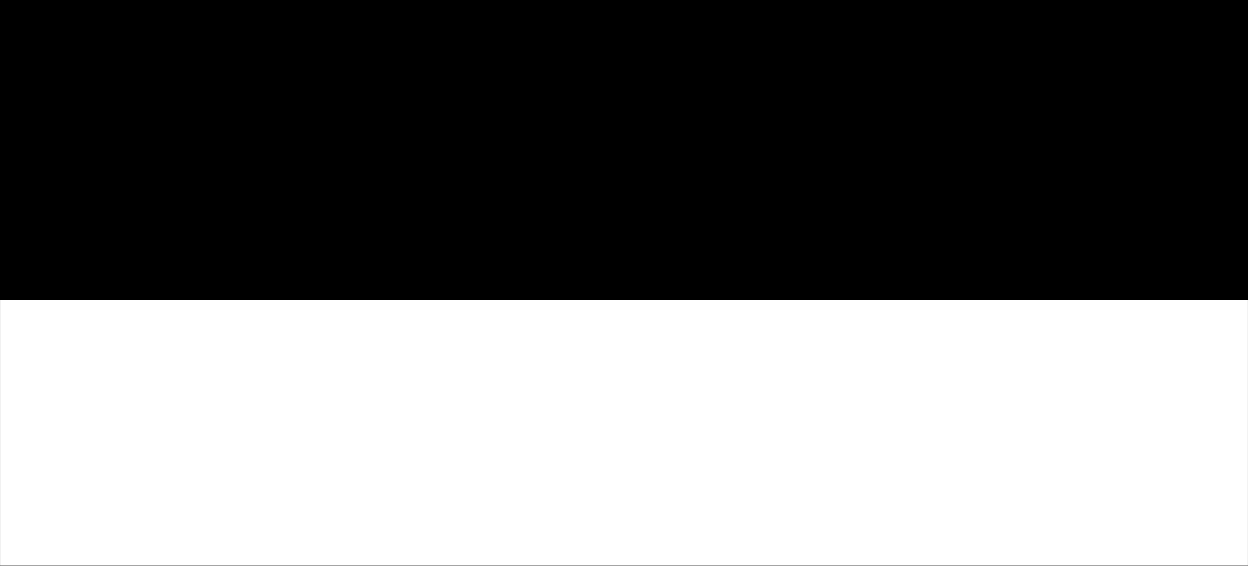

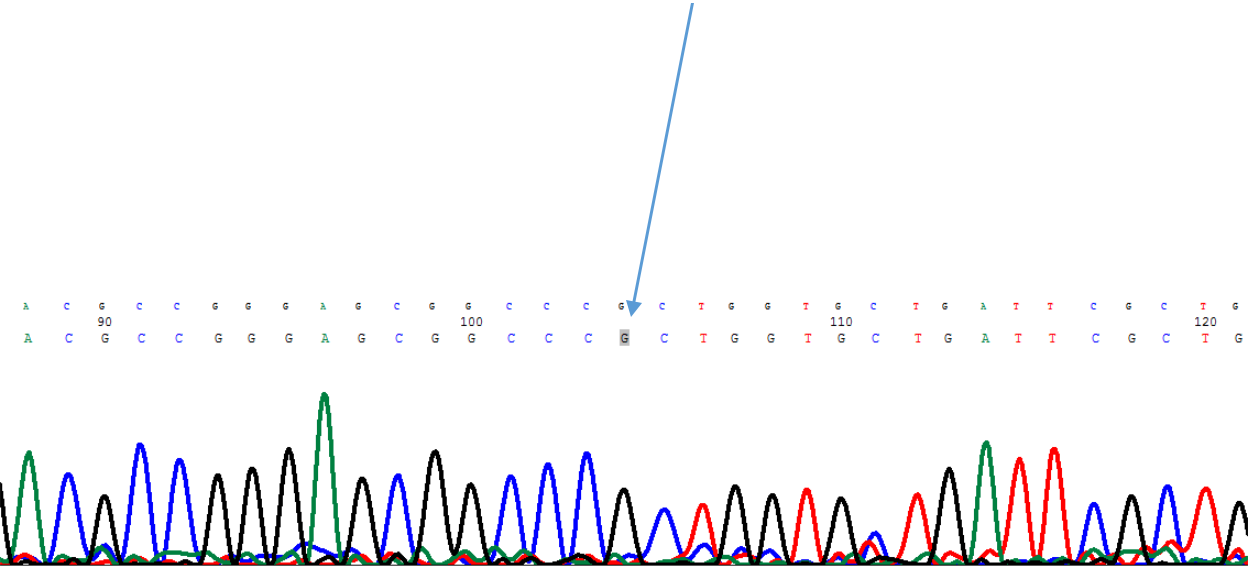


************************************************************

ABCB6 GGGGGCCGGCCCTCGCATCTCTCCCTACGTGCTGCAGCTGCTTCTGGCCACACTTCAGGC

6524_AB2_ GGGGGCCGGCCCTCGCATCTCTCCCTACGTGCTGCAGCTGCTTCTGGCCACACTTCAGGC

6524_AB1_ GGGGGCCGGCCCTCGCATCTCTCCCTACGTGCTGCAGCTGCTTCTGGCCACACTTCAGGC

************************************************************

ABCB6 GGCGCTGCCCCTGGCCGGCCTGGCTGGCCGGGTGGGCACTGCCCGGGGGGCCCCACTGCC

6524_AB2_ GGCGCTGCCCCTGGCCGGCCTGGCTGGCCGGGTGGGCACTGCCCGGGGGGCCCCACTGCC

6524_AB1_ GGCGCTGCCCCTGGCCGGCCTGGCTGGCCGGGTGGGCACTGCCCGGGGGGCCCCACTGCC

************************************************************

**CONCLUSION**

| **Sample Number** | **Gene** | **Mutation** | **BASE Call** | **Result** |
| --- | --- | --- | --- | --- |
| A | ZFHX4 | G12411T L4137F | T | G12411T detected |
| B | GJA8_Cx50 | c.649G>A (Val196Met) | G | c.649G>A Not detected |
| F | FGFR2 | S267P (T-C) | T | mutation not detected. |
| F | FGFR2 | C278F (G-T) | G | mutation not detected. |
| F | FGFR2 | Q289P (A-C) | A | mutation not detected. |
| F | FGFR2 | C342S (G-C) | G | mutation not detected. |
| F | FGFR2 | C342Y (G-A) | G | mutation not detected. |
| F | FGFR2 | C342W (C-G) | G | mutation not detected. |
| F | FGFR2 | A344A (G-A) | G | mutation not detected. |
| F | FGFR2 | S347C (C-G) | C | mutation not detected. |
| D | STRA6 | T>C P.Y374C | C | T>C P.Y374C detected |
| D | STRA6 | A>T P.L152M | T | mutation not detected. |
| D | CRYBA4 | C>T P.R25W | C | Mutation not detected |
| D | OTX2 | p. Gln104 X | C | p. Gln104 X Not detected |
| D | OTX2 | p. Gln106 His | C | p. Gln106 His Not detected |
| D | OTX2 | p. Thr186 Fs<frame shift | G | p. Thr186 Fs<frame shift Not detected |
| C | ABCB6 | p. Ala 57 Thr G^🡪^A | G | p. Ala 57 Thr G^🡪^A Not detected |
